# Mutational burden of XPNPEP3 leads to defects in mitochondrial complex I and cilia in NPHPL1

**DOI:** 10.1016/j.isci.2023.107446

**Published:** 2023-07-23

**Authors:** Lingxiao Tong, Jia Rao, Chenxi Yang, Jie Xu, Yijun Lu, Yuchen Zhang, Xiaohui Cang, Shanshan Xie, Jianhua Mao, Pingping Jiang

**Affiliations:** 1 Department of Nephrology, The Children’s Hospital, Zhejiang University School of Medicine and National Clinical Research Center for Child Health, Hangzhou, China; 2 Department of Nephrology, Children’s Hospital of Fudan University, National Pediatric Medical Center of China, Shanghai, China; 3 Department of Human Genetics, Zhejiang University School of Medicine, Hangzhou, China; 4 Zhejiang Key Laboratory for Neonatal Diseases, The Children’s Hospital of Zhejiang University School of Medicine, Hangzhou, China

**Keywords:** disease, human Genetics, cell biology

## Abstract

Nephronophthisis-like nephropathy-1 (NPHPL1) is a rare ciliopathy, caused by mutations of *XPNPEP3*. Despite a well-described monogenic etiology, the pathogenesis of XPNPEP3 associated with mitochondrial and ciliary function remains elusive. Here, we identified novel compound heterozygous mutations in NPHPL1 patients with renal lesion only or with extra bone cysts together. Patient-derived lymphoblasts carrying c.634G>A and c.761G>T together exhibit elevated mitochondrial XPNPEP3 levels via the reduction of mRNA degradation, leading to mitochondrial dysfunction in both urine tubular epithelial cells and lymphoblasts from patient. Mitochondrial XPNPEP3 was co-immunoprecipitated with respiratory chain complex I and was required for the stability and activity of complex I. Deletion of Xpnpep3 in mice resulted in lower activity of complex I, elongated primary cilium, and predisposition to tubular dilation and fibrosis under stress. Our findings provide valuable insights into the mitochondrial functions involved in the pathogenesis of NPHP.

## Highlights

• Novel compound heterozygous mutations of XPNPEP3 are identified in NPHPL1 patients
• XPNPEP3 is required for the stability of mitochondrial complex I
• Xpnpep3-KO mice replicate some NPHP-like phenotypes with elongated primary cilium

## Graphical abstract

**Figure:**
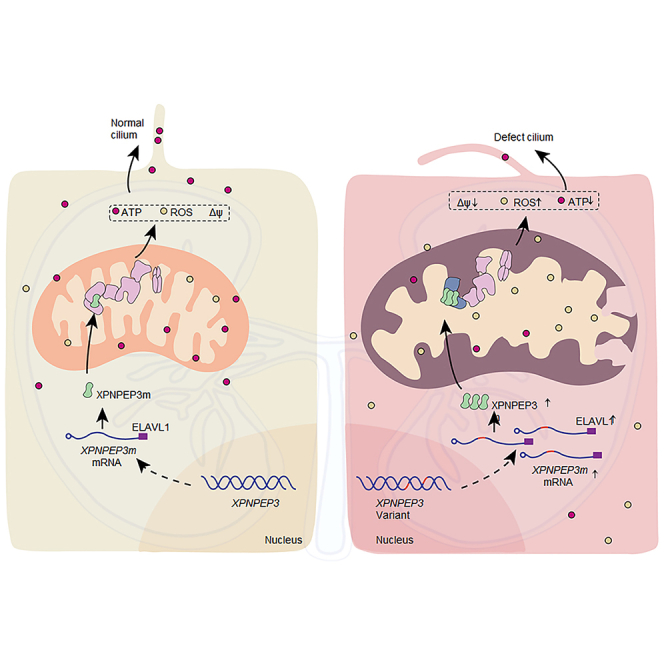

## Introduction

Nephronophthisis (NPHP, OMIM: #256100) is a tubulointerstitial, autosomal recessive cystic kidney disease and the most frequent genetic cause of pediatric end-stage kidney disease (ESKD). NPHP is characterized by polyuria, polydipsia, increased echogenicity on ultrasonography and tubulointerstitial alterations in histopathology, such as tubular atrophy, thickening or thinning of the tubular membrane, interstitial fibrosis, and cyst formation.^1^ As the clinical presentation of NPHP is frequently nonspecific, molecular genetic analysis has currently emerged as an essential tool for the accurate clinical diagnosis of NPHP. Currently, 26 different NPHP genes have been identified.^2^ Most of these genes are expressed in the primary cilia, basal bodies, or centrosomes of renal epithelial cells. They are involved in ciliary structure and function or intraflagellar transport (IFT) and contribute to the wide phenotypic spectrum in NPHP.^1^^,^^3^ NPHP has thereby been included in “ciliopathies” or “NPHP-related ciliopathies (NPHP-RCs)”. To date, multiple signaling pathways have been implicated in the pathogenesis of NPHP, such as Hedgehog (Hh),^4^ canonical Wnt/β-catenin,^5^^,^^6^ Hippo,^7^^,^^8^ and DNA damage response (DDR).^9^^,^^10^ However, the majority of molecular mechanisms are cilia dependent, yet the underlying molecular etiology and pathogenesis remain incomplete.

Distinct from ciliary proteins, XPNPEP3 is absent from the primary cilia and basal body complex. One form of XPNPEP3 is predicted to have a mitochondrial localization (XPNPEP3m), while the other is cytosolic (XPNPEP3c).^11^^,^^12^Mutations of XPNPEP3 cause nephronophthisis-like nephropathy-1 (NPHPL1, OMIM: #613159), which is exceedingly rare.^13^^,^^14^ Only 6 patients from 3 consanguineous families have been reported, and these patients harbored one of 3 homozygous mutations of *XPNPEP3*. O’Toole et al. identified a homozygous splice-site mutation (c.1357G>T; p.G453C) with moderate renal insufficiency between 20 and 29 years of age, and another homozygous 4-bp deletion (c.931_934 delAACA; p.N311Lfs∗5) that resulted in a truncated protein in the catalytic domain, which associated with childhood ESKD by 8–9 years of age and severe extrarenal manifestations.^13^ Alizadeh et al. found a homozygous insertion mutation, c.719_720insA; p.Q241Tfs∗13, that also resulted in a truncated protein in a 13-year-old girl with NPHPL1 and overall growth failure.^14^ Additionally, a single heterozygous variant c.463C>T (p.R155W) was reported as having unknown significance in an NPHP patient.^15^ Obviously, different mutations of XPNPEP3 give rise to diverse phenotypes depending on the mutant alleles. Mechanistically, XPNPEP3 is a member of the aminopeptidase P family that specifically removes the N-terminal amino acid from peptides with a Pro residue at the second position. It has been proposed that XPNPEP3c cleaves several ciliary proteins that cause renal cysts, such as LRRC50,^16^ ALMS1,^17^ and CEP290/NPHP6.^18^ The loss of activity of XPNPEP3-mediated enzymatic cleavage of these ciliary proteins may contribute to NPHPL1.^12^^,^^13^ Yeast intermediate-cleavage peptidase (Icp55), an orthologous to human XPNPEP3, plays a role in mitochondrial protein processing and protein stabilization that involves components of the respiratory chain complex.^19^^,^^20^ Moreover, ciliogenesis is coupled to the abundance and function of mitochondria.^21^ However, as a mitochondrial protein, the pathogenic role of XPNPEP3 in mitochondrial and ciliary dysfunction is incompletely understood.

Here, we identified two unrelated NPHPL1 patients harboring compound heterozygous mutations of *XPNPEP3* by whole-exome sequencing (WES). One patient harbored c.634G>A and c.761G>T mutations and presented with hyperechogenic kidneys. The other patient harbored c.-87C>T and c.1261C>G mutations and presented with renal cysts, proteinuria, hematuria, and bone cysts. To investigate the underlying molecular mechanism, we developed several models of XPNPEP3 adaptation in cells and mice and explored the impacts of c.634G>A and c.761G>T on XPNPEP3, mitochondria, and the primary cilium to better understand the role of XPNPEP3 in NPHPL1 and the connection between mitochondria and ciliopathy.

## Results

### Identification of XPNPEP3 variants associated with NPHPL1

**Figure 1:**
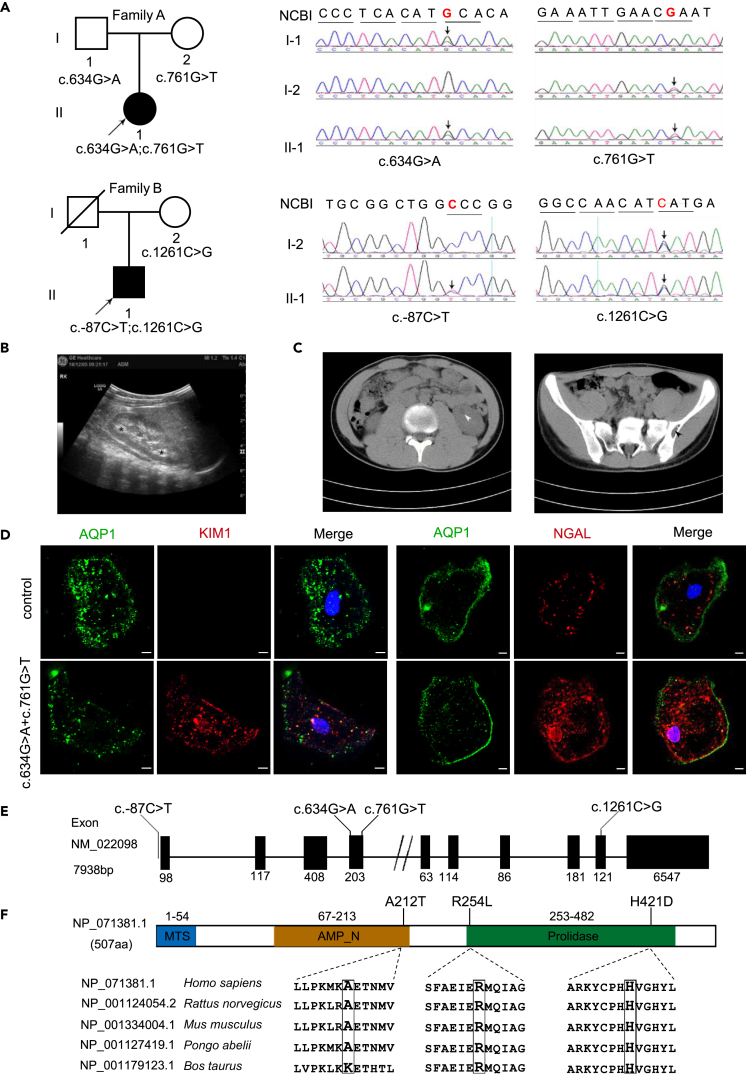
Patients with NPHPL and compound heterozygous mutations of X*PNPEP3* (A) Pedigrees of Family A and Family B. Family A harbors variants c.634G>C and c.761G>T; Family B harbors variants c.-87C>T and c.1261C>G. Arrows indicate nucleotide changes. Square for males; circles for females; solid for patients with NPHPL1; slash for deceased individuals. (B) Ultrasound image of patient A-II-1. Asterisks for medulla. (C) Abdominal CT image of patient B-II-1, showing the left kidney cyst (left panel) and left ilium cystic foci (right panel). Arrowheads indicate the foci. (D) Immunofluorescence analysis of KIM1 and NGAL in tubular epithelial cells. AQP1, aquaporin 1, a water channel protein located in the kidney proximal tubule. Bar: 5μm. (E) Schematic of *XPNPEP3* gene variants. The *XPNPEP3* gene is located on chromosome 22q. Both the c.634G>C and c.761G>T variants in Family A are located in exon 4, while the c.-87C>T and c.1261C>G variants in Family B are distributed in the noncoding region and exon 9, respectively. (F) Conservation analysis of XPNPEP3 proteins. Linear domain organization of the XPNPEP3 protein showing the p.A212 residue located in the AMP_N (aminopeptidase) domain and the p.R254 residue and p.H421 residue in the prolidase domain. MTS, mitochondrial target signal.

The two unrelated probands, an 11-month-old female infant (A-II-1) and a 13-year-old boy (B-II-1), were diagnosed with NPHPL1 via clinical and genetic testing (Figure 1A). Patient A was born normally at 38 weeks of gestation from healthy nonconsanguineous Chinese parents. She underwent a left nephrectomy for Wilms’ tumor likely at 10 months of age in another hospital. Renal ultrasonography showed irregular echogenicity at right with enlarged medulla contours (Figure 1B). During the 3-year follow-up, no proteinuria, hematuria, or renal insufficiency occurred. Patient B presented with multiple renal cysts in both kidneys at 10 years of age. Moreover, cystic foci, 22∗8 mm, were detected in the left ilium (Figure 1C). He developed proteinuria at 11 years of age. At this age, his 24-h urine protein excretion was 0.32–0.48 g, and this increased to 1.3 g at 12 years of age. Hyperuricemia (408 μmol/L) and an elevation in serum cystatin C levels (1.23 mg/L, reference range 0.55–1.03 mg/L) were recorded recently (Table 1). Kidney injury was identified in tubular epithelial cells from urine samples of patient A as biopsy/nephrectomy samples were unavailable. Compared to an age-matched control, the expression levels of kidney injury molecule 1 (KIM1) and neutrophil gelatinase-associated lipocalin (NGAL) were higher (Figure 1D). The father of patient B died of an unknown cause, and the mother was phenotypically normal. Grandparents from the mother and father side as well as siblings were all free of nephropathy at present, while grandmother of patient A had passed away from uremia two years ago.

**Table 1:** *XPNPEP3* variants in 2 unrelated families with an NPHP-like kidney disease

Patients	A-II-1	B-II-1
Nucleotide alteration, exon	634G>A and 761G>T,4	−87C>T,1; and 1261C>G,9
Protein change	p.A212T; p.R254L	non-coding; p.H421D
Allele in affected individuals (segregation)	compound het (het in F, M)	compound het (het in F, M)
Renal function (at age, yr)		
24-h urine protein excretion	WNL	1.3g (12)
serum uric acid	WNL	408 μmol/L (13)
serum cystatin C levels	WNL	1.23 mg/L (13)
Renal ultrasound (age, yr)	↑EG,no cysts(10mons)	↑EG, cysts(10)
Extrarenal manifestations	none	cystic foci in left ilium

Het, heterozygous; F, father; M, mother; WNL, within normal limits; ↑EG, increased echogenicity.

Biallelic variants of *XPNPEP3* were identified in each patient by WES, and confirmed by Sanger sequencing, whose segregation pattern fits a recessive mode of inheritance. Two missense variants, c.634G>A, (chromosome 22: 41282361G>A, hg 19) and c.761G>T (chromosome 22: 41282488G>T, hg 19) in exon 4 were found in patient A. These mutations result in an alanine-to-threonine substitution at position 212 (p.A212T) in the aminopeptidase domain and an arginine-to-leucine substitution at position 254 (p.R254L) in the prolidase domain. The c.-87C>T (chromosome 22: 41253099C>T, hg 19) variants, which are upstream of the gene, and the c.1261 C>G (chromosome 22: 41320390C>G, hg 19) variant in exon 9 were identified in patient B; the latter results in a histidine-to-aspartic acid substitution at position 421 (p.H421D) in the prolidase domain of XPNPEP3 (Figures 1E and 1F). No other causative genes associated with Wilms’ tumor, NPHP, or NPHP-like nephropathy were detected. The c.634G>A variant was absent in public databases, including gnomAD; but the c.-87C>T, c.761G>T, and c.1261 C>G variants occurred in gnomAD with frequencies of 0.006%, 0.01%, and 0.004%, respectively. To date, this is the first report of these 4 variants in NPHPL1 cases. In the ClinVar database, the c.-87C>T variant was interpreted to be of an uncertain significance. The other three novel missense variants are conserved in mammals. Additionally, a damaging effect on protein function was predicted with c. 1261C>G, but a probably benign effect was indicated for c.634G>A or c.761G>T alone (Table S1). However, no significant alteration in the conformation or stability was disrupted by either of the A212T and R254L mutations alone or in combination in the protein by molecular dynamics simulation analysis (Figure S1). These findings elicited questions regarding how the combination of two “benign” mutations led to infantile NPHPL1 and the molecular mechanism underlying the role of XPNPEP3 and mitochondria in the pathogenesis of NPHPL1.

### Mutant XPNPEP3 increases protein expression by increasing mRNA stability

**Figure 2:**
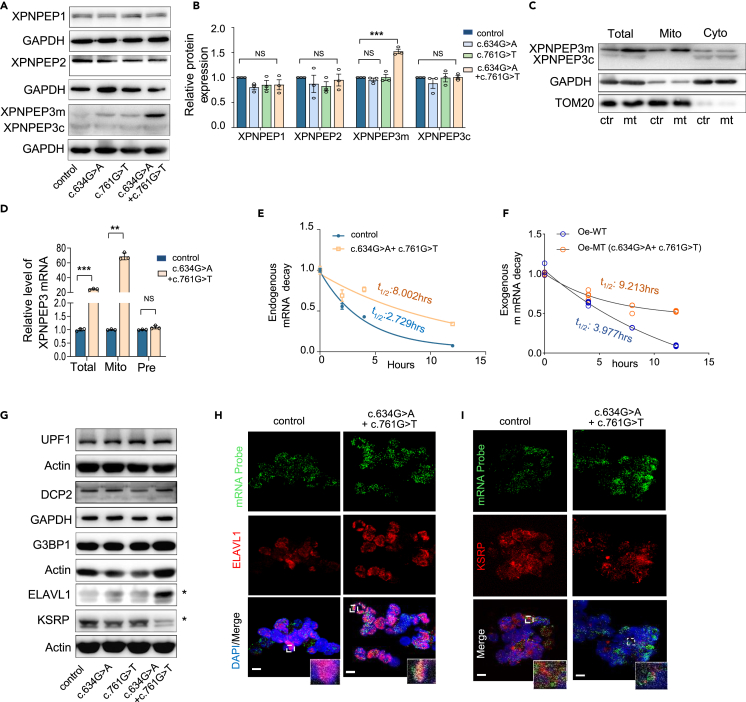
Elevated expression of mitochondrial XPNPEP3 via a reduction of mRNA degradation (A) Western blot analysis of XPNPEP1, XPNPEP2, and XPNPEP3 in lymphoblast cell lines derived from Family A and an age-matched control to patient A. GAPDH was used as a loading control. (B) Quantification of protein expression. (C) Subcellular expression of XPNPEP3 by WB with anti-XPNPEP3, TOM20 (mitochondrial marker), and GAPDH (cytosol marker) antibodies. Total, total cell lysate; Mito, mitochondria; Cyto, cytosol; ctr, control cells; mt, cells with *XPNPEP3* variants c.634G>A and c.761G>T. (D) Reverse transcriptase quantitative PCR (RT-qPCR) analysis of XPNPEP3. Total, mRNA level of *XPNPEP3*; Mito, mRNA levels of mitochondrial *XPNPEP3* isoform; Pre, pre-mRNA levels of XPNPEP3. (E) Fitted exponential decay curves of XPNPEP3m mRNA levels in lymphoblast cells. Transcription was blocked by 10 μg/mL actinomycin D. (F) Fitted exponential decay curves of XPNPEP3m mRNA levels *in vitro*. Oe-WT, wild type of XPNPEP3m transfected back into *XPNPEP3*-knockout HK-2 cell lines; Oe-MT, XPNPEP3m carrying c.634G>A and c.761G>T transfected back into *XPNPEP3*-knockout HK-2 cell lines. (G) Protein levels of UPF1, DCP2, G3BP1, and ELAVL1 detected by western blotting. UPF1, regulator of nonsense transcripts 1; DCP2, m7GpppN-mRNA hydrolase; G3BP1, GTPase-activating protein-binding protein 1; ELAVL1, ELAV-like protein 1; KSRP, KH-type splicing regulatory protein. (H) Colocalization of XPNPEP3m RNA with ELAVL1 by combined RNA FISH and immunofluorescence. Bar, 2 μm. (I) Colocalization of XPNPEP3m RNA with KSRP by combined RNA FISH and immunofluorescence. Bar, 2 μm. Data were shown as the mean ± SD of triplicates. Student’s t test was performed between two groups and one-way ANOVA was performed among four groups. ∗, p < 0.05; ∗∗, p < 0.01; ∗∗∗, p < 0.001. NS, not significant.

To investigate the impacts of the c.634G>A and c.761G>T variants of XPNPEP3 on etiology, patient-derived lymphoblasts from Family A (individuals carrying c.634G>A or c.761G>T solely or the patient with compound heterozygous variants), as well as a genetically unrelated control, were used to generate an *ex vivo* model to investigate the underlying mechanisms of this disease. We performed western blotting (WB) to examine the expression levels of XPNPEP3 and two other isozymes of the protein family, the cytosolic enzymes XPNPEP1 and XPNPEP2, both of which have been identified as key inactivators of bradykinin peptide.^22^^,^^23^^,^^24^ As illustrated in Figures 2A and 2B, patient-derived lymphoblasts showed no change in the levels of XPNPEP1, XPNPEP2, and cytosolic XPNPEP3 (XPNPEP3c), but an approximately 50% increase in the expression of mitochondrial XPNPEP3 (XPNPEP3m) (p < 0.001). To confirm the different levels of the two isoforms of XPNPEP3 in the mitochondria and cytosol, a fraction of mitochondria was roughly isolated from whole cell and analyzed with the remaining cytosol in parallel, compared to the whole-cell lysates as a control. As expected, the upper bands from mitochondria in the mutant cell line were upregulated compared with the controls, indicating that the mitochondrial isoform was predominant. However, no difference was found in XPNPEP3_C_ between the control and mutant cell lines (Figure 2C). These findings indicate that the expression of XPNPEP3m is increased in compound heterozygotes for c.634G>A and c.761G>T, which is contrast to the reported findings of a common reduction in mutant protein expression by missense or nonsense mutations.^25^

To determine whether the increase in XPNPEP3m arose from its increased transcription or from mRNA stability, we measured the mature and precursor mRNA (pre-mRNA) levels of *XPNPEP3* in mutant and control cell lines by quantitative real-time PCR (RT-qPCR). Noticeably, the levels of total mutant *XPNPEP3* and *XPNPEP3m* mRNA were increased approximately 24-fold and 69-fold compared to the wild type, respectively (Figure 2D). However, the expression level of mutant pre-mRNA remained unchanged compared to the wild type, indicating that the higher level of mature mutant mRNA was dependent on the enhanced stability of mRNA. A similar finding was obtained from the transcription inhibition assay by actinomycin D, which showed that the mutant transcript had a longer half-life in mRNA degradation (8.002 h) than the wild type (2.729 h) (Figure 2E). Furthermore, we constructed *XPNPEP3*-knockout (KO) HK-2 cell lines by CRISPR/Cas9 (Figure S2) and transfected them with plasmids carrying wild type (Oe-WT) and mutant XPNPEP3m (Oe-MT) to test the level of mRNA stability *in vitro*. Consistently, the exogenous mutant XPNPEP3m displayed (t_1/2:_ 9.213 h) a lower level of degradation by mRNA decay than the wild type (t_1/2_:3.977 h) (Figure 2F). Together, these findings strongly support that the compound heterozygous mutations in *XPNPEP3* gave rise to a higher level of XPNPEP3m protein because of a reduction in mutant mRNA decay.

The control of mRNA stability has an important role in gene expression, which is mediated through messenger RNA surveillance pathways, including nonsense-mediated mRNA decay (NMD), *cis*-acting elements, or *trans*-acting factors.^26^^,^^27^^,^^28^ We therefore examined the expression levels of NMD-related proteins and several RNA binding proteins (RBPs) to determine which factor was involved. As shown in Figures 2G and 2H, the expression levels of UPF1, the key effector of NMD,^28^ DCP2, the decapping enzyme affecting the stability of mRNAs,^29^ and G3BP1, which binds the 3′-UTRs of mRNAs,^30^ were comparable in cell lines. However, the stabilizing factor ELAVL1 was significantly upregulated in patient-derived cells, and the KH-type splicing regulatory protein (KSRP), a destabilizing AU-rich element (ARE)-binding protein, was significantly downregulated (Figure 2G). No difference was observed in lymphoblasts carrying either c.634G>A or c.761G>T relative to the controls. We further conducted fluorescence *in situ* hybridization (FISH) of *XPNPEP3m* mRNA and immunofluorescence staining for ELAVL1 to determine their conditions. The patient-derived lymphoblasts presented higher levels of *XPNPEP3m* mRNA and ELAVL1 and more colocalized granules than the controls (Figure 2H). Conversely, few colocalized granules between *XPNPEP3m* mRNA and KSRP were observed in patient cells elsewhere (Figure 2I).^31^ These data indicated that ELAVL1 was responsible for the enhanced stability of mutant mRNA and the increased levels of XPNPEP3 protein caused by the compound heterozygous mutations. However, the mechanism by which ELAVL1 and KSRP protected mutant XPNPEP3 RNA from degradation requires further investigate.

### Mutant XPNPEP3m perturbs mitochondrial function and morphology and leads to cell apoptosis

**Figure 3:**
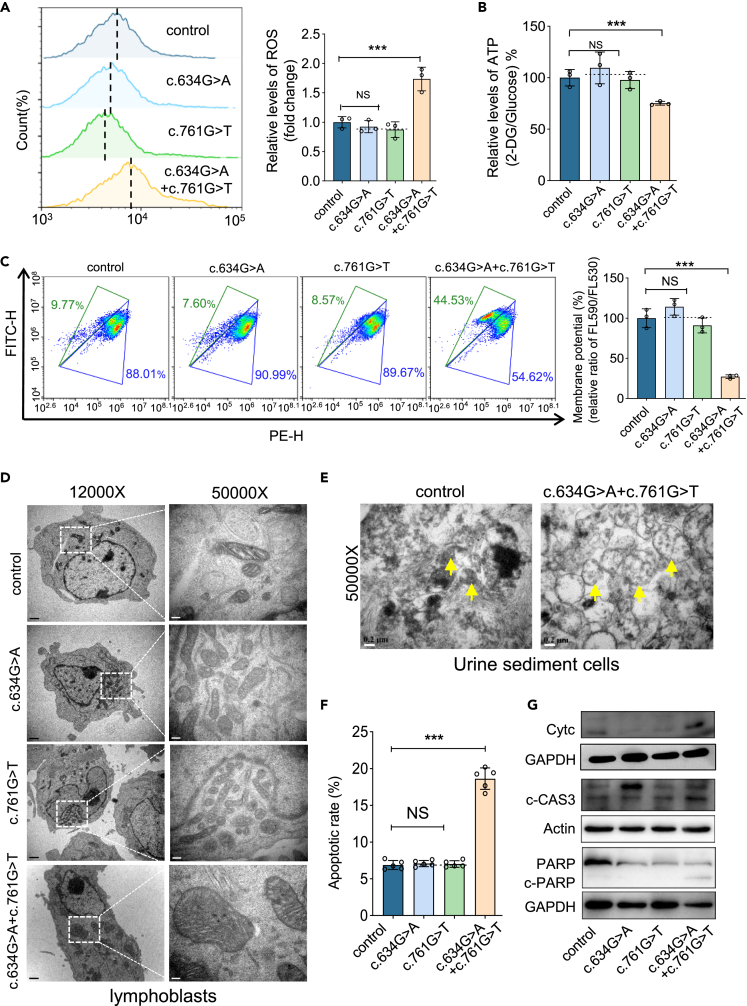
XPNPEP3m perturbs mitochondrial function and morphology and leads to cell apoptosis (A) Mitochondrial ROS in lymphoblasts, measured by flow cytometry with MitoSOX (n = 3). (B) The relative ratio of mitochondrial ATP production, detected by luciferase assay. Cells were incubated with 2-deoxy-D-glucose plus pyruvate (n = 3). (C) The measurement of mitochondrial membrane potential (MMP) by flow cytometry with JC-10 mitochondrial probe (n = 3). The odds ratio (%) of JC-10 fluorescence intensities present as Ex/Em (FL590/FL530) between the mutant and the control. (D) Mitochondrial morphology in lymphoblasts by TEM. Bar (12000X), 1 μm; Bar (50000X), 0.2 μm. (E) Mitochondrial morphology in tubular epithelial cells by TEM, collected from patient A. Arrow, mitochondria. Bar, 0.2 μm. (F) The proportion of apoptotic cells, performed by flow cytometry using combined annexin-V and PI staining (n = 5). (G) Western blot analysis of apoptosis-associated proteins with antibodies against Cytc (cytochrome *c*), c-CAS3 (cleaved caspase 3), PARP (poly [ADP-ribose] polymerase 1), c-PARP (cleaved PARP). Data are shown as the mean ± SD of triplicates at least. One-way ANOVA was performed among four groups. ∗, p < 0.05; ∗∗, p < 0.01; ∗∗∗, p < 0.001. NS, not significant.

XPNPEP3 is suggested to be a mitochondrial matrix protein.^11^ Our exogenous XPNPEP3m tagged with hemagglutinin (HA) was expressed in HK-2 cells. It exclusively localized in mitochondria and was absent from the cilium, as indicated by immunofluorescence (Figures S3A and S3B). Considering the key role of mitochondria in kidney injury,^32^ we investigated whether this resident protein had an effect on mitochondrial function and morphology. As shown in Figure 3A, the production of mitochondrial reactive oxygen species (ROS) in mutant cells with c.634G>A and c.761G>T was increased to 173% (p < 0.001), as tracked by a MitoSOX indicator. In contrast, the mitochondrial ATP amount in mutant cells was reduced by 25% (p < 0.01), relative to the controls (Figure 3B). A similar finding was observed in the mitochondrial membrane potential (MMP), in which patient lymphoblasts had a lower level of MMP, with a mean value of 27% (p < 0.001) relative to the controls (Figure 3C). However, no differences of ROS, ATP, or MMP were detected in cells harboring only one of the variants (Figures S3C and S3D). In addition to mitochondrial dysfunction, abnormal mitochondrial morphology is usually observed in samples from patients with renal disease.^32^ As expected, swollen mitochondria were observed in both lymphoblasts and renal tubular epithelial cells from urine samples of patient A by transmission electron microscopy (TEM) (Figures 3D and 3E). Mitochondrial XPNPEP3 was reported to have an anti-apoptotic function.^33^ Consistently, a higher level of apoptosis in lymphoblasts from the patient was observed with a mean value of 19.4% (p < 0.001) compared to that of 6.3% in controls using annexin V and propidium iodide double staining (Figures 3F and S3E). This was further confirmed by increased levels of the apoptosis-related proteins cytochrome *c*, cleaved caspase 3 (c-Cas3), and cleaved poly (ADP-ribose) polymerase-1 (c-PARP) (Figure 3G). Moreover, aberrant XPNPEP3m with dysfunctional mitochondria led defects in cell proliferation and migration (Figure S4). Thus, our data indicate that mutant XPNPEP3m leads to mitochondrial dysfunction and subsequent cell apoptosis.

### XPNPEP3m is required for the activity and stability of mitochondrial complex I

**Figure 4:**
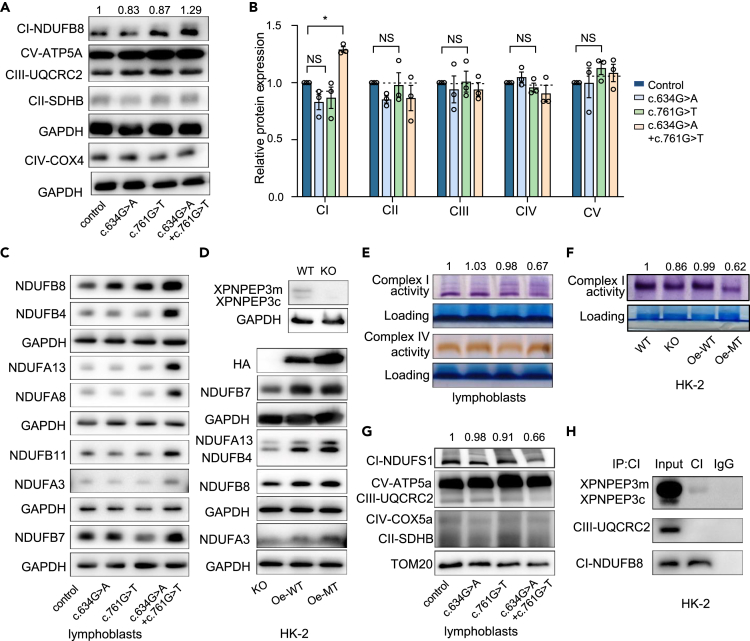
Mutant XPNPEP3m decreases the activity and stability of mitochondrial complex I (A) Western blot analysis of subunits of respiratory complexes I-V in lymphoblasts by OXPHOS antibody cocktail: NDUFB8 (complex I, CI), SDHB (complex II, CII), UQCRC2 (complex III, CIII), COXIV (complex IV, CIV) and ATP5A (complex V, CV). GAPDH, as a loading control. (B) Quantification of protein levels of respiratory complexes I-V. (C) Western blot analyses of subunits of complex I in lymphoblasts with antibodies against NDUFB8, NDUFB4, NDUFA13, NDUFA8, NDUFB11, NDUFA3, and NDUFB7. GAPDH, as a loading control. (D) Western blot analyses of subunits of complex I in HK-2 cell lines. WT, cells with wild type XPNPEP3; KO, XPNPEP3 knockout HK-2 cells; Oe-WT, wild type XPNPEP3m transfected back into KO cell lines; Oe-MT, XPNPEP3m carrying c.634G>A and c.761G>T transfected back into KO cell lines. (E) In-gel activity of complexes I and IV in lymphoblasts by native PAGE. (F) In-gel activity of complexes I in different HK-2 cell lines, descript as above. (G) Stability of fully assembled complex I in lymphoblasts by BN-PAGE analysis, hybridized with antibody cocktail specific for subunits of OXPHOS complex and TOM20 as a loading control. (H) XPNPEP3m present in complex I. Mitochondrial lysates were subjected to immunoprecipitation by complex I capture antibody and immunoblotted with anti-XPNPEP3, UQCRC2 for complex III, and NDUFB8 for complex I. Data were shown as the mean ± SD of triplicates. One-way ANOVA was performed among four groups. ∗, p < 0.05; ∗∗, p < 0.01; ∗∗∗, p < 0.001. NS, not significant.

Since the mutant XPNPEP3m in patient A resulted in mitochondrial dysfunction, we questioned which mitochondrial respiratory chain complex was involved and how it impacted this process. Individuals with frameshift mutations in *XPNPEP3* have shown functional defects in mitochondrial respiratory chain complex 1 (RCC1),^13^ suggesting a potential molecular mechanism underlying XPNPEP3m and complex I. We thereby tested whole protein levels of complexes of the respiratory chain first. As shown in Figures 4A and 4B, patient-derived cells showed upregulated expression of mitochondrial complex I as it was 129% (p < 0.05) of the average values in control cells. However, cells with solely c.634G>A or c.761G>T lacked changes in complex I. Furthermore, there were no significant differences observed in complexes II, III, IV, and V between control and mutant cell lines, as determined by the total OXPHOS antibody cocktail. Consistently, increased expression levels of complex I subunits, NDUFB8, NDUFB7, NDUFB4, NDUFA13, NDUFA8, and NDUFA3, were detected in patient lymphoblasts (Figure 4C), indicating that the mitochondrial dysfunction induced by XPNPEP3m was mostly due to the disturbed complex I. To investigate whether XPNPEP3m potentially affects the activity and/or stability of complex I, we transfected HA-tagged wild-type or mutant XPNPEP3m back into the XPNPEP3-KO HK-2 cell line. As expected, overexpression of either WT (Oe-WT) or mutant XPNPEP3m (Oe-MT) increased the subunit levels of complex I (Figure 4D), verifying the involvement of XPNPEP3m in the function of complex I. Subsequently, a 33% (p < 0.01) reduction in activity of complex I was illustrated by clear native polyacrylamide gel electrophoresis, and alterations in activity of complex IV were absent in various lymphoblast cell lines (Figure 4E). Furthermore, similar impaired activity of complex I was present in XPNPEP3-KO cells (86%, p < 0.01). This was then restored by the overexpression of WT-XPNPEP3m, but not by the mutant XPNPEP3m (62%, p < 0.01) (Figure 4F). These findings confirm that XPNPEP3m is required for the activity of mitochondrial complex I, and the mutant protein impairs the activity of complex I. Considering that the impaired activity of complex I may result from its instability, we thereby assessed the stability of complex I by blue native polyacrylamide gel electrophoresis (BN-PAGE). As shown in Figure 4G, the level of assembled complex I in lymphoblast cells was 66% (p < 0.01) of the mean values measured in the controls. Interestingly, we found that XPNPEP3m was captured as a component of complex I by immunoprecipitation of total cellular extracts from HK-2 cells (Figure 4H), which is in agreement with previous observation that yeast Icp55 attached to the mitochondrial inner membrane.^20^ However, we could not detect a certain subunit of complex I that physically interacted with XPNPEP3, suggesting that XPNPEP3 might act as a cofactor for its assembly. Taken together, these findings demonstrate that XPNPEP3m is required for the activity and stability of mitochondrial complex I, and the mutant XPNPEP3m is responsible for the deficiency of complex I, which in turn leads to mitochondrial dysfunction.

### *Mitochondrial XPNPEP3 accounts for* ciliary defects

**Figure 5:**
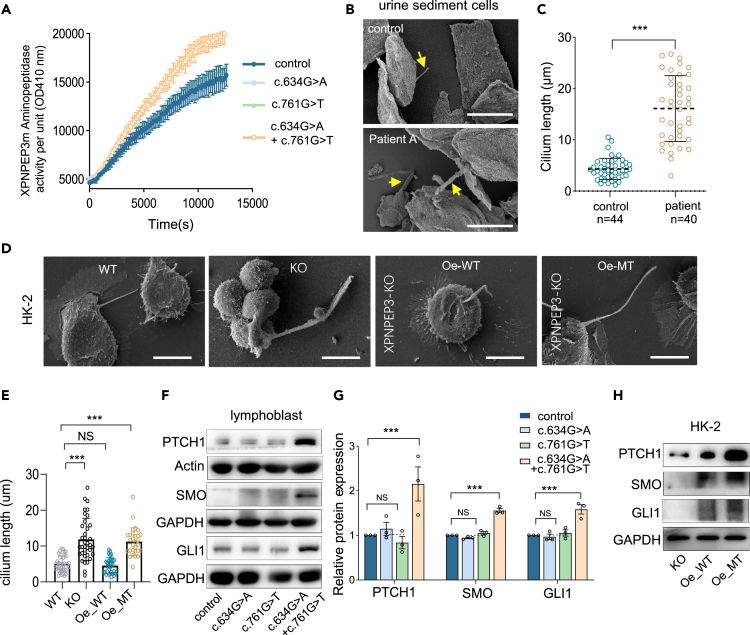
Mitochondrial XPNPEP3 accounts for ciliary defects (A) Aminopeptidase enzymatic activity of XPNPEP3m, measured with substrate peptide H-Lys (Abz)-Pro-Pro-pNA by bioluminescence assay (n = 3). (B) Cilia morphology in tubular epithelial cells by SEM. Arrows, cilia; Bar:20 μm. (C) Graphical representation of cilia length in control (n = 44) and patient A (n = 40) tubular epithelial cells, with a mean value of of 4.3 μm and 16.1 μm (p < 0.001) respectively. (D) Cilia morphology in HK-2 cells by SEM, cultured without FBS for 48 h. Bar:10 μm. (E) Graphical representation of cilia length in HK-2 cell lines. WT, 5 μm (n = 38); KO, 11.9 μm (n = 35), Oe-WT,4.6 μm (n = 31); Oe-MT, 11.2 μm (n = 29). (F) Western blot analysis for ciliary function in lymphocytes and with antibodies against proteins involved in Hh signaling pathway. PTCH (protein patched homolog 1), SMO (smoothened, frizzled class receptor), GLI1 (GLI family zinc finger), and GAPDH or Actin as a loading control. (G) Relative levels of PTCH, GLI1 and SMO (n = 3). (H) Western blot analysis for ciliary function in HK-2 KO cells with wild type or mutant XPNPEP3m transfected back. Data were expressed as the means ± SD (n ≥ 3). Student’s t test was performed between two groups and one-way ANOVA was performed among four groups. ∗∗∗, p < 0.001.

It was previously suggested that the loss of proper cleavage of ciliary proteins by defective cytosolic XPNPEP3 contributes to NPHPL1.^13^ To evaluate the impact of mutations on enzyme activity, we tested X-pro-aminopeptidase activity using isolated mitochondria from lymphoblast cells and incubated them with the fluorogenic substrate H-Lys (Abz)-Pro-Pro-pNA as described elsewhere.^33^ Compared to controls, the overactivation of X-pro-aminopeptidase subjected to XPNPEP3m was highly present in lymphoblasts from patient A (Figure 5A). Considering that NPHPL1 is characterized by ciliary defects,^3^ we investigated whether the mutant XPNPEP3m affected ciliary morphology and/or function. We first checked the morphology of the primary cilium in renal tubular epithelial cells from urine samples of patient A, and age-matched control samples, because patient biopsy/nephrectomy samples were unavailable. By scanning electron microscopy (SEM) analysis, notably elongated cilia were observed, with a mean value of 16.1 μm (p < 0.001) in urine sediment cells, compared to controls (4.3 μm) (Figures 5B and 5C). This phenotype was also observed in HK-2 cells. As illustrated in Figures 5D and 5E, XPNPEP3m deficiency led to longer primary cilia (11.9 μm, p < 0.001) than that of controls (5.0 μm), which was restored when wild-type XPNPEP3m was expressed in KO cells. These findings reveal that mitochondrial XPNPEP3 is responsible for the alteration of cilia. Moreover, elongated cilia were also observed when mutant XPNPEP3m was transfected back into KO cells, with a mean value of 11.2 μm (p < 0.001) in length. These data reveal that either elevated XPNPEP3m or XPNPEP3 deficiency elongates cilia length, eliciting the influence of mitochondrial dysfunction on ciliary defects.

Considering that the Hh signaling pathway is frequently disrupted by defects in ciliary function, we next explored the effect of mutant XPNPEP3m on ciliary function by checking the mRNA levels of PTCH1 (patched 1) and GLI1 (GLI family zinc finger 1), and their protein levels as well as SMO (smoothened, fizzled class receptor) as reported elsewhere.^3^ The expression of PTCH1 (215%, p < 0.001), SMO (155%, p < 0.001), and GLI1 (158%, p < 0.001) was upregulated in lymphoblasts from patient compared with that in cells from either control individuals or carriers (Figures 5F and 5G). Consistently, significant increases of mRNA of PTCH1 and GLI1 were observed when mutant *XPNPEP3m* was expressed in HK-2 KO cells compared to that in WT-*XPNPEP3m* cells (Figure S5). Additionally, increased proteins of PTCH1, GLI1, and SMO were exhibited in HK-2 cells with mutant *XPNPEP3m* expressed, whereas SMO and GLI1 were distinctly absent in HK-2-KO cells (Figure 5H). Together, these data indicate that XPNPEP3m accounts for not only ciliary anomalies but also ciliary function defects.

### Xpnpep3-KO mice replicate NPHPL phenotypes with mitochondrial dysfunction and cilia defects

**Figure 6:**
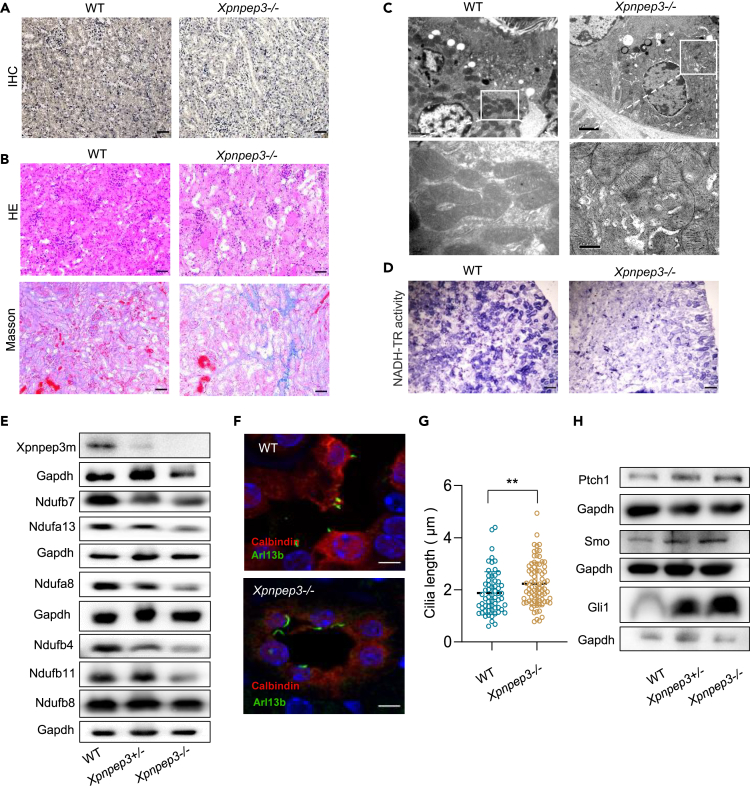
Mitochondrial dysfunction and ciliary defects in kidney tissues of *Xpnpep3*^*−/−*^ mice (A) Identification of Xpnpep3 knockout mice by IHC assay. Bar, 50μm. (B) HE and Masson staining of kidney sections in WT and *Xpnpep3*^*−/−*^ mice under acute kidney injury, Bar, 50μm. (C) Mitochondrial morphology in tubular epithelial cells in mice by TEM. Bar,2 μm. (D) NADH-TR enzymatic activity in kidney sections, Bar,100 μm. (E) Western blot analysis for subunits of mitochondria complex I from kidney tissues. (F) Measurement of ciliary length of kidney tubular epithelial cells from WT and *Xpnpep3*^*−/−*^ mice, using antibodies against ciliary protein Arl13b, and Calbindin, a marker for kidney distal tubule and the proximal part of the collecting ducts. Bar, 5μm. (G) Statistical analysis of the primary cilium length. WT, 1.87 μm in length (n = 61); *Xpnpep3*^*−/−*^, 2.23μm in length (n = 81). (H) Western blot analysis for Hh pathway protein Ptch1, Smo, and Gli1 in renal tissues. Mice were used at age of 8 weeks. Data were expressed as the means ± SD. Student ‘s t test was performed between two groups. ∗, p < 0.05. ∗∗, p < 0.01. ∗∗∗, p < 0.001.

As mentioned earlier, disturbing the homeostasis of XPNPEP3, by either abolishing or increasing its expression, led to an elongated primary cilium and its dysfunction in cells. In contrast to the overactivation of XPNPEP3, the inactivation of XPNPEP3 was usually caused by damaging homozygous mutations, as previously described in patients with kidney injury.^13^ We then wondered whether the loss of function of XPNPEP3m still led to mitochondrial dysfunction and cilia defects *in vivo*. As samples from patient B were unavailable, we constructed *Xpnpep3*-KO mice using the CRISPR/Cas9 system, resulting in a c.732delG mutation in exon 4 (Figure S6) and substantial deficiency of the Xpnpep3 protein in kidney tissue from homozygous mice, as shown by IHC staining (Figure 6A). At the age of 16 weeks, there was slight tubular epithelial cell shedding and cast formation in the kidney sections of *Xpnpep3*^*−/−*^ mice (Figure S6). Strikingly, with the application of cisplatin (CDDP) to simulate mouse models of acute kidney injury (AKI), *XPNPEP3*^*−/−*^CDDP-AKI mice had obvious tubular dilation in HE staining and fibrosis in Masson staining compared with WT-CDDP-AKI mice (Figure 6B). In addition, ruptured mitochondria were observed in *Xpnpep3*^*−/−*^mice by TEM (Figure 6C). Furthermore, the activity of mitochondrial complex I was significantly decreased in kidney tissues of *Xpnpep3*^*−/−*^ mice, as shown by staining analysis of NADH-tetrazolium reductase (NADH-TR) (Figure 6D). Accordingly, the subunits of mitochondrial complex I in *Xpnpep3*^*−/−*^ mouse kidney were reduced, which was similar to the results observed in lymphoblasts and HK-2 cell lines (Figure 6E). To determine if alteration in the cilia length of kidney tubular epithelial cells exists between WT and *Xpnpep3*^*−/−*^ mice, ciliary protein Arl13b was labeled with Calbindin, a marker for kidney distal tubule and the proximal part of the collecting ducts. As expected, *Xpnpep3*^*−/−*^ mice had longer cilia than control mice (p < 0.01) (Figures 6F and 6G), but without marked change in the number of ciliated cells (Figure S7). Moreover, ciliary defects in *Xpnpep3*^*−/−*^ mice were also evidenced by higher mRNA levels of *Ptch1* and *Gli1*(Figure S5), and higher protein levels of Ptch1, Gli1, and Smo than those in WT mice (Figure 6H). Thus, the KO of *Xpnpep3*^*−/−*^ *in vivo* recapitulated the NPHPL-like phenotype with mitochondrial dysfunction and ciliary defects.

## Discussion

NPHP disorders display significant genetic and phenotypic heterogeneity, and *XPNPEP3* is a rare causative gene of NPHPL1 that only occurs in a few patients worldwide. A full understanding of XPNPEP3, especially its function in mitochondria and its functional relationship to cilia, is incomplete. Herein, we expanded the molecular spectrum of *XPNPEP3* with compound heterozygous mutations in two NPHPL1 patients and revealed that patient-derived lymphoblasts carrying c.634G>A and c.761G>T exhibited elevated level of mitochondrial XPNPEP3 via a reduction in its mRNA degradation by recruiting more ELAVL1. We found XPNPEP3 in mitochondrial complex I, and the elevated levels of XPNPEP3m in mitochondria reduced the activity and stability of mitochondrial complex I in both patient lymphoblasts and HK-2 cell lines. This subsequently led to mitochondrial dysfunction and cell apoptosis, with diminished ATP products, decreased MMP levels, and increased ROS generation. Either increased or decreased expression of mitochondrial XPNPEP3 accounted for the elongated primary cilium and dysregulated Hh signaling in both endogenous and exogenous XPNPEP3 assays. *Xpnpep3-KO* mice displayed decreased activity of complex I and longer cilia and were predisposed to tubular dilation and fibrosis under stress.

Recently, components of the respiratory chain complex have been shown to be the substrates of Icp55 in yeast, including subunits of ATPase, ATP3, ATP11, and ATP16.^20^ However, neither subunits nor its assembly factors of complex V were changed, but the interplay between XPNPEP3m and mitochondrial complex I was elucidated in this study. Despite its aminopeptidase activity in the mitochondrial matrix,^19^ XPNPEP3m acted as a component in complex I and was required for its stability and activity. This would explain the complex I deficiency that was observed in a muscle biopsy from a previous NPHPL1 patient.^13^ Interestingly, Icp55 has been isolated from mitochondrial membranes.^20^ Both the elevated expression of XPNPEP3 induced by the compound heterozygous mutations in patient and Xpnpep3 deficiency in mice led to deficiency of complex I, which in turn affected mitochondrial morphology and function. The dysfunction of mitochondria subsequently led to apoptotic death and impaired cell proliferation and motility, which directly influence renal function.^34^^,^^35^ Consistently, aberrant XPNPEP3m with dysfunctional mitochondria increased apoptosis and defects in cell proliferation and migration (Figures 3 and S4).

Cilia defects are a hallmark of NPHP. Fewer or shortened cilia were reported in deficiency of ciliary proteins, such as TTC30 A/B,^36^ ANKS6,^8^ and IFT144.^37^ Conversely, longer cilia have been previously described in fibroblasts or kidney biopsies from some IFT172,^38^ TTC26^39^ or ARL16^40^ patients. There was no significant difference in the number of ciliated cells observed between mutant urine sediment cells and controls from patient, and between *Xpnpep3*-KO and WT mice (Figure S7). However, longer primary cilia with abnormal Hh signaling were evoked by the aberrant expression of XPNPEP3 in either patient urine sediment cells or kidney tubular epithelial cells of *Xpnpep3*-KO mice, emphasizing the major role of XPNPEP3 homeostasis in mitochondrial function and ciliopathy. Given that only XPNPEP3m was increased by the mutations, the elongated cilia in the patient seem to be independent of the cytosolic XPNPEP3. Furthermore, longer cilia were induced when mutant XPNPEP3m was overexpressed compared to the WT control, indicating that mitochondrial function is associated with ciliogenesis. Certainly, mitochondrial dysfunction plays an important role in the progression of kidney diseases and ciliopathy. Bae JE et al.^41^ found that treatment with the mitochondrial respiratory complex-1 inhibitor rotenone stimulated ciliogenesis in both SH-SY5Y and RPE cells, as evidenced by an increase in ciliary length and prevalence. However, a striking loss of rotenone-induced ciliogenesis was observed in SH-SY5Y cells treated with the ROS scavenger N-acetyl cysteine (NAC).^41^ Moreover, reduced ATP levels also contribute to the elongation of cilia.^21^ Knocking out KIF19A, a kinesin located at the tip of cilia that depolymerizes ciliary microtubules from the plus end in an ATP-dependent fashion, consequently led to longer cilia in different organ systems of the mouse.^42^

Considering the impacts of XPNPEP3 on mitochondria and cilia, extrarenal manifestations, such as cardiomyopathy and seizures as demonstrated in one NPHPL1 kindred,^13^ that may be caused by mitochondrial dysfunction were supposed to be observed. However, the kidney-only phenotype in patient A suggested that the c.634G>A and c.761G>T mutations were functionally less destructive than homozygous truncating mutations, which was consistent with our allele prediction on protein function. However, patient A was only an 11-month-old infant, and follow-up is needed for efficient intervention and monitoring of disease. Patient B, a 13-year-old boy, harboring c.-87C>T and c.1261 C>G mutations, presented with hematuria, proteinuria, renal cysts, and an extra bone cyst in the left ilium. Bone abnormalities have not been described in other NPHPL1 patients but have appeared in some patients with ADPKD.^43^ The manifestations of the two patients were much milder than those of affected individuals identified previously, indicating the possibility that diverse phenotypes depended on the function of mutant alleles. Strikingly, a mild kidney phenotype was also observed in our *Xpnpep3*-KO mice as slight tubular epithelial cell shedding and cast formation in the kidney sections were observed in *Xpnpep3*^*−/−*^ mice untill 16 weeks of age. The compensatory effects of its paralogs XPNPEP1 and XPNPEP2, which highly expressed in the kidney as metallopeptidases, may also contribute to this phenotype. XPNPEP1 was proposed to process ciliary cystogenic proteins to prevent the disease phenotype caused by XPNPEP3 mutations.^19^ However, when stimulated with cisplatin injection, *Xpnpep3*^*−/−*^ mice soon developed tubular dilation and fibrosis in the kidney, indicating that other factors, such as oligogenes, modifier genes, or even environmental factors, may contribute to NPHPL1 phenotypes synergistically. Certainly, the lack of an obvious kidney phenotype in mouse models has been reported in other Nphp-KO mice, such as Nphp1^44^ and Nphp11,^45^ indicating the compensation of other genes and the complexity of the pathogenesis.

In summary, we have identified defects in mitochondrial complex I and primary cilia in NPHPL1 patients with compound heterozygous mutations of *XPNPEP3*, in which the combination of c.634G>A and c.761G>T increases the expression of mitochondrial XPNPEP3 by reduction of its mRNA degradation. As a component, XPNPEP3m is required for the activity and stability of mitochondrial complex I. Aberrant expression of XPNPEP3 results in mitochondrial dysfunction and ciliary defects *in vivo and in vitro*. *Xpnpep3*-KO mice replicate some NPHPL phenotypes with elongated cilia. These insights will be essential to improve the understanding of the pathogenesis of NPHP and other ciliopathies.

### Limitations of the study

Here we identified novel heterozygous compound mutations in XPNPEP3 in two patients with NPHPL1. The protein level of mitochondrial XPNPEP3 was elevated from the combination of c.634G>A and c.761G>T. The enhanced stability of mRNA was determined by endogenous and exogenous mRNA decay assays and was stabilized by higher level of ELAVL1 and reduced KSRP. However, the question that how ELAVL1 and KSRP recruited for protecting the mutant XPNPEP3 RNA from degradation requires further investigation.

## STAR★Methods

### Key resources table

**Table:** 

REAGENT or RESOURCE	SOURCE	IDENTIFIER
**Antibodies**		

XPNPEP1	Abcam	Cat#ab235324
XPNPEP2	Proteintech	Cat#25945-1-AP; RRID:AB_2880305
AQP1	Proteintech	Cat#66805-1-Ig; RRID:AB_2882148
KIM1	Abcam	Cat#ab228973; RRID:AB_2915918
NGAL	Proteintech	Cat#26991-1-AP; RRID:AB_2880715
XPNPEP3	Genetex	Cat#GTX105541; RRID:AB_1952597
GAPDH	Abcam	Cat# ab8245; RRID:AB_2107448
TOM20	Abclonal	Cat#A19403; RRID:AB_2862646
UPF1	Proteintech	Cat#23379-1-AP; RRID:AB_11232421
DCP2	Abclonal	Cat#A8282; RRID:AB_2769123
G3BP1	Proteintech	Cat#13057-2-AP; RRID:AB_2232034
ELAVL1	Proteintech	Cat#11910-1-AP; RRID:AB_11182183
KSRP	Proteintech	Cat#55409-1-AP; RRID:AB_11182170
β-actin	Proteintech	Cat#66009-1-Ig; RRID:AB_2687938
Total OXPHOS Rodent WB Antibody Cocktail	Abcam	Cat#ab110413; RRID:AB_2629281
COX-IV	Abcam	Cat#ab16056; RRID:AB_443304
NDUFB8	Proteintech	Cat#14794-1-AP; RRID:AB_2150970
NDUFB4	Abclonal	Cat#A13820; RRID:AB_2861696
NDUFB7	Proteintech	Cat#14912-1-AP; RRID:AB_2235903
NDUFA13	Abclonal	Cat#A3782; RRID:AB_2863140
NDUFA8	Abclonal	Cat#A12118; RRID:AB_2759008
NDUFB11	Proteintech	Cat#16720-1-AP; RRID:AB_2298378
NDUFA3	Proteintech	Cat#17257-1-AP; RRID:AB_2150631
NDUFS1	Proteintech	Cat#12444-1-AP; RRID:AB_2282657
ATP5a	Abcam	Cat#ab176569; RRID:AB_2801536
UQCRC2	Proteintech	Cat#14742-1-AP; RRID:AB_2241442
COX5a	Abcam	Cat#ab181226
SDHB	Abcam	Cat#ab178423; RRID:AB_2861366
Cytochrosome c	Abclonal	Cat#A4912; RRID:AB_2863387
Caspase3	CST	Cat#9662
PARP	CST	Cat#9532
PTCH1	Abclonal	Cat#A0826; RRID:AB_2757415
SMO	Proteintech	Cat#66851-1-Ig; RRID:AB_2882191
GLI1	Proteintech	Cat#66905-1-Ig; RRID:AB_2882232
Anti-HA	Abclonal	Cat#AE008; RRID:AB_2770404
Calbindin	Proteintech	Cat#66394-1-Ig; RRID:AB_2881769
ARL13B	Proteintech	Cat#17711-1-AP; RRID:AB_2060867
Complex I Immunocapture	Abcam	Cat#ab109798; RRID:AB_10862214
Goat anti mouse IgG(H + L) (HRP)	Beyotime	Cat# A0216; RRID:AB_2860575
Goat anti rabbit IgG(H + L) (HRP)	Beyotime	Cat# A0208; RRID:AB_2892644
Alexa Fluor 488 goat anti-mouse IgG	Abcam	Cat#ab150113; RRID:AB_2576208
Alexa Fluor 594 goat anti-rabbit IgG	Abcam	Cat#ab150080; RRID:AB_2650602
Anti-IgG	Proteintech	Cat#B900620; RRID:AB_2883054

**Chemicals, peptides, and recombinant proteins**		

DAPI	Sigma	Cat#D9542
Mitotracker	CST	Cat#9082
Mitomycin C	Selleck	Cat#BIO-000001
CCK8	Yeasen	Cat#40203ES60
Trizol reagent	Sangon	Cat#B511311
Hieff *trans* transfection reagent	Yeasen	Cat#40802ES03
Puromycin	Gibco	Cat#A1113802
DDM	MedChemExpress	Cat#HY-128974
Actinomycin D	Sigma	Cat#A9415
H-Lys(Abz)-Pro-Pro-pNA	Bachem	Cat#4027668
FCCP	Sigma	Cat# C2920-10MG
NBT	Merck	Cat#298-83-9
NADH	Merck	Cat#606-68-8
DAB	Merck	Cat#91-95-2
cytochrome *c*	Merck	Cat#C3483

**Critical commercial assays**		

MitoSOX™ Red Mitochondrial Superoxide Indicator, for live-cell imaging	Invitrogen	Cat# M36008
JC-10 Mitochondrial Membrane Potential Assay Kit (Flow Cytometry)	Abcam	Cat#ab112133
CellTiter-Glo Luminescent Cell Viability Assay	Promega	Cat#G7571
Reverse transcription kit	Takara	Cat#6110A
Cell apoptosis assay kit	Beyotime	Cat#C1062L
EDU flow cytometry kit	Sangon Biotech	Cat#E607204

**Experimental models: Cell lines**		

Lymphocytes	This paper	N/A
Tubular epithelial cells	This paper	N/A
HK-2	National Collection of Authenticated Cell Cultures	Cat# SCSP-511

**Experimental models: Organisms/strains**		

Mouse:C56BL/6J	Jackson Laboratory	Cat# 000664
Mouse:*Xpnpep3*KO	This paper	N/A

**Oligonucleotides**		

Primers	This paper	See Table S2

**Recombinant DNA**		

pRK-3HA-*XPNPEP3m*	This paper	N/A
pRK-3HA-*XPNPEP3m*^c.634G>A andc.761G>T^	This paper	N/A
pRK-3HA-*XPNPEP3m*^*c*.*1261C>G*^	This paper	N/A

**Software and algorithms**		

GraphPad Prism v8.0	Graphpad Software	https://www.graphpad.com
ImageJ/Fiji	Schindelin et al.^46^	https://imagej.net/software/fifiji/

### Resource availability

#### Lead contact

Further information and requests for resources and reagents should be directed to and will be fulfilled by the lead contact, Pingping Jiang (ppjang@zju.edu.cn).

#### Materials availability

This study did not generate new unique reagents; All requests for resources and reagents should be directed to the lead contact author.

### Experimental model and study participant details

#### Families and subjects

The study design adhered to the tenets of the Declaration of Helsinki. All family members provided written informed consent, using a form approved from the Research Ethics Committees, the Children’s Hospital of Zhejiang University School of Medicine, before blood collection. Clinical evaluations consisted of comprehensive history, physical examination, standard renal function tests, and renal imaging were performed to identify personal or family medical histories of renal impairment or other clinical abnormalities.

#### Cell culture and plasmids

Lymphocytes derived from members of Family A and Family B and were immortalized by transformation with the Epstein-Barr virus, as described elsewhere.^47^ Tubular epithelial cells were obtained from urine samples of patient A harboring XPNPEP3 c.634G>A and c.761G>T variant and an unrelated age-matched individual. Cells were grown in RPMI 1640 medium (Thermo Fisher Scientific), supplemented with 10% FBS (Gibco). lentiCRISPRv2 plasmid (Addgene; 52961) with oligos of sgRNAs was transfected into HK-2 to generate XPNPEP3 knockout cell lines using Hieff TransTM Liposomal Transfection Reagent according to the manufacture protocol. Cells were selected using puromycin (7 μg/mL) for 24 h, and then incubated with the standard medium. Cell clones was confirmed via PCR and sequencing. The pcDNA3.1(+) and pRK-3HA (CMV-MCS-IRES-SV40-ampicillin) vectors were used to overexpress the wild and mutant XPNPEP3m cDNA. Variants of c.634G>A and c.761G>T in cDNA were generated by site-directed mutagenesis with its corresponding primers (Table S2). In brief, PCR amplification was performed with pRK-3HA-XPNPEP3m as a template using KOD-Plus-Neo (Toyobo,#F1066K).The PCR product was digested by DpnI (Takara, #1235S) at 37°C for 30 min, following the bacterial transformation and amplification. The plasmids were isolated by an endotoxin free plasmid isolation kit (EndoFree Plasmid Mini Kit, Easydo,#DR0202050).The variant was validated by PCR sequencing. Primers were listed in Table S2.

#### Generation of XPNPEP3m-KO mice and genotyping

All animal care protocols and experimental procedures were approved by the Animal Care and Use Committee of Zhejiang University School of Medicine. *XPNPEP3m*-KO mice were generated using the CRISPR/Cas9 system by targeting genomic RNA (AAGGAGACTTAACCAGCCTT) for *XPNPEP3* by Biogle (Changzhou, China). The genotypes for the XPNPEP3^+/−^, XPNPEP3^−/−^mice were confirmed by PCR amplification and direct sequencing.

#### Mouse models of acute kidney injury (AKI)

Intraperitoneal injection of 10 mg/kg cisplatin (CDDP) was applied for the generation of CDDP-AKI mouse model for 72 h in 4–6 weeks’ mice.

### Method details

#### Sequencing and genetic data analysis

Genomic DNA of peripheral blood was extracted using a DNA Blood Mini kit (Qiagen) according to the kit instructions. A library of whole exomes was built using Roche Nimble Gen Seq EZ Exome Enrichment Kit V2.0 and Seq EZ Exome Enrichment Kit V2.0 capture probes (Roche). High-throughput sequencing was performed by 150-bp paired-end sequencing with Illumina NovaSeq 6000 series sequencer (PE150). The clean data were aligned to the NCBI human reference genome (hg19) using Burrows-Wheeler Aligner-Maximal Exact Match (BWA-MEM) algorithm.^48^ Variants were called using HaplotypeCaller (https://gatk.broadinstitute.org/)^49^ and annotated with databases such as Online Mendelian Inheritance in Man (OMIM), Exome Aggregation Consortium (ExAC), and predicted by online analysis programs. Mutations of *XPNPEP3* (GenBank: NC_000022.11) identified in probands and patients were validated by Sanger sequencing. Primers were listed in Table S2.

#### Western blot assay

The cells were collected and lysed on ice with 50 mM Tris-HCl (pH 7.4), 150 mM NaCl, Triton X-100, 1% sodium deoxycholate, 0.1% SDS and 10% PMSF. Per lane 20 μg proteins were subjected to an 8%–15% sodium dodecyl sulfate-polyacrylamide (SDS-PAGE) gel and transferred on PVDF membrances (Millipore). The membranes were blocked in 5% milk and incubed with primary antibodies overnight at 4°C. The HRP-conjugated anti-rabbit or anti-mouse IgG was used as secondary antibodies. Signals were detected with ECL solution and imaged with a Clinx-Chemi-Capture system (Clinx Science Instruments). ImageJ was used to quantify the bands. Primary antibodies were listed in Table S3.

#### Immunofluorescence staining

Tubular epithelial cells from urine sediments or HK-2 cells seeded on coverslips were fixed in 4% paraformaldehyde for 30 min, permeabilized in 0.2% Triton X-100 for 15 min, and blocked in 5% bovine serum albumin (BSA) for 1 h at RT. Cells were incubated with primary antibodies overnight at 4°C, co-incubated with fluorescent secondary antibodies for 1 h, and finally counterstained with DAPI for 8 min. Images were taken by Olympus Fluoview FV1000 and analyzed with ImageJ. The following primary antibodies: Fluoview FV1000 and analyzed with ImageJ. The following primary antibodies: anti-AQP1, anti-KIM1, anti-NGAL, anti-ELAVL1, anti-Calbindin, anti-ARL13B, anti-HA and secondary antibodies: Alexa Fluor 488 goat anti-mouse IgG and Alexa Fluor 594 goat anti-rabbit IgG, were used and listed in Table S3.

#### RNA FISH

Biotin-labeled *XPNPEP3m* mRNA probes were prepared following RiboTM RNAmax-T7 Transcription Kit instructions. Lymphocytes were fixed in 4% formaldehyde and permeabilized with 0.5% Triton X-100 for 5 min, washed with PBS three times, dehydrated and rehydrated with a gradient of ethanol. Then cells were probed with the *XPNPEP3m* mRNA FISH probes, washed, stained with DAPI, and mounted using the anti-fade medium. Images were captured by Olympus Fluoview FV1000 and analyzed with ImageJ.

#### Immunoprecipitation (IP) assay

For the immunoprecipitation (IP) analysis, HK-2 cells were collected and lysed on ice for 30 min in lysis buffer with with 25 mM Tris-HCl, 150 mM NaCl, 1% NP-40 and 0.5% N-Dodecyl-β-D-maltoside (DDM) in pH 7.4. The whole-cell lysate was collected by centrifuged at 20,000 g for 10 min at 4°C and incubated with 10 μL of beads (cross-linked to a monoclonal antibody, anti-Complex I Immunocapture (1:10) or control IgG (1:200) overnight at 4°C with rotation. Beads were washed 4 times at 4°C with cold PBS buffer, eluted by boiling in 1× SDS loading buffer for 5 min, and then subjected to SDS-PAGE for Western blot analyses with relevant antibodies.

#### Quantification of mRNA half-life

Cell was treated with actinomycin D (10 μg/mL) to block transcription, and collected at 0, 2, 4 and 12 h after treatment, then followed by RNA extraction and qRT-PCR. The housekeeping gene *18sRNA* was used as control as previously described.^50^ The level of mRNA half-lives was calculated with fitted nonlinear exponential decay curves.

#### Quantitative real-time PCR assays (qRT-PCR)

The level of mRNA expression was performed with qRT-PCR assays. Total RNA was extracted using Trizol Reagents. cDNA was prepared by amplifying 500 ng of RNA with a reverse transcription kit. Quantitative PCR was performed using LightCycler 480 SYBR Green I Master following manufacturer’s instructions and normalized on the basis of *GAPDH* mRNA or *18sRNA*. Primers were listed in Table S1.

#### BN-PAGE and in-gel activity assays

Mitochondrial proteins were isolated from cells as detailed elsewhere.^51^ Samples containing 30 μg of mitochondrial proteins were separated on 3%–11% gradient Native PAGE Bis-Tris gel and run at 150 V in dark-blue cathode buffer for an hour, and then 250 V in the light-blue running buffer at 4°C for 1.5 h. The native PAGE gels were then prewashed in an ice-cold transfer buffer (25 mM Tris, 192 mM glycine, 20% methanol) and transferred onto the PVDF membrane at 30 V overnight for immunoblotting. After blocking with 5% BSA for 1 h, the membrane was incubated with primary antibodies at 4°C overnight. Images were captured and quantified as described above.

The enzymatic activities of OXPHOS complexes I and IV were measured by in-gel activity assay as detailed elsewhere.^52^ In brief, samples containing 20 μg of mitochondrial proteins were loaded and run at 150V in light-blue cathode buffer for 1 h, then at 250 V in clear cathode buffer. The native gels were prewashed in ice-cold water and then incubated with the substrates of complex I (1 mM Tris-HCl, pH 7.4; 1 mg/mL nitroblue tetrazolium; 0.1 mg/mL NADH) and complex IV (50 mM phosphate buffer, Ph7.4; 0.5 mg/mL diaminobenzidine; 1 mg/mL cytochrome c) at 4°C overnight. After the reaction was stopped with 10% acetic acid, the gels were washed extensively in water and scanned to assess the activities of the respiratory chain complexes.

#### Measurement of XPNPEP3m enzymatic activity

Samples (2 μL) containing 30 μg of mitochondrial proteins were added into 198 μL substrates in 96-well black plate, which was included 5 μM H-Lys(Abz)-Pro-Pro-pNA, 0.5 mmol/L Mncl2.4H_2_O, and 0.1 mmol/L HEPES. The mixture was incubated at 37°C for 3 h and the fluorescence (Ex/Em = 320/410 nm) intensity was measured at 5 min intervals using the Synergy H1 (BioTek).

#### Transmission or scanning electron microscopy

Mitochondria morphology was visualized with transmission electron microscopy. Cells were fixed in 2.5% glutaraldehyde, post-fixed in 1% OsO_4_, dehydrated in a graded series of ethanol solution, washed in acetone, and embedded in resin mixture. Sections were obtained by EM UC7 ultratome (Leica, Germany), and then stained by uranyl acetate and lead citrate. Images were taken by H-7650 transmission electron microscope (Hitachi, Japan). The ultrastructure of cilium in cells was observed using scanning electron microscopy. After dehydrated through an ethanol series and washed with pure ethanol, cell samples were inserted into HCP-2 critical point dryer (Hitachi, Japan) until dry, coated with gold-palladium in E−1010 ion sputter (Hitachi, Japan) for 4–5 min and observed in the SU-8010 scanning electron microscope (Hitachi, Japan).

#### ATP measurements

Cellular and mitochondrial, Cellular and mitochondrial ATP levels were measured by the CellTiter-Glo Luminescent Cell Viability Assay, following the manufacturer’s instructions.

#### Assessment of mitochondrial membrane potential, assessment of mitochondrial membrane potential

The levels of mitochondrial membrane potential (MMP) were measured by JC-10 Mitochondrial Membrane Potential Assay Kit according to the manufacturer’s recommendations. In brief, 1×10^6^ lymphocytes were harvested, resuspended with 200 μL 1× JC-10 assay buffer, and then incubated at 37°C for 30 min. Cells were analyzed by the NovoCyte flow cytometer with excitation (Ex)/emission (Em) = 490/525 and 490/590 nm.

#### Measurement of ROS production

MitoSOX assay was used for the measurement of mitochondrial ROS generation. Briefly, 1 × 10^6^ lymphocytes treated with or without 10 μM of antimycin A were resuspended in PBS supplemented with 5 μM of MitoSOX, and then incubated at 37°C for 20 min. Cells were analyzed with excitation at 488 nm and emission 529 nm with the NovoCyte flow cytometer.

#### Measurement of NADH-tetrazolium reductase (NADH-TR) enzymatic activity

NADH incubation solution (200μL) containing 0.2M Tris-HCl, 200 μg Nitro blue tetrazolium, and 160 μg NADH was put onto 10μm frozen sectioned slides of the kidney at 37°C for 30min. The stained slides then were rinsed in distilled water, dehydrated, cleared, and mounted. Images were taken by Leica DM4000B-M.

### Quantification and statistical analysis

Data were analyzed using GraphPad Prism 8.0 and expressed as means ± SD with at least three independent experiments. Statistical analysis was performed by two-tailed Student’s *t* test (with 95% confidence interval) for two groups and ordinary one-way analysis of variance (ANOVA) followed by the Fisher LSD post hoc test (assuming equal variances) or Tamhane’s T2 post hoc test (without the assumption of equal variances) for three or more groups. A *p* value < 0.05 was considered statistically significant (∗, p < 0.05; ∗∗, p < 0.01; ∗∗∗, p < 0.001).

## Data Availability

All data reported in this paper will be shared by the lead contact upon request. This paper does not report original code. Any additional information required to reanalyze the data reported in this paper is available from the lead contact upon request.

## Supplemental information

Document S1. Figures S1–S7 and Tables S1–S3

Datas S1–S19
