# Supplementary material for: Mutational burden of XPNPEP3 leads to defects in mitochondrial complex I and cilia in NPHPL1
Source: iScience. 2023 Jul 23;26(8):107446. doi: 10.1016/j.isci.2023.107446 (PMC10432713; doi:10.1016/j.isci.2023.107446)
Supplement: Document S1. Figures S1–S7 and Tables S1–S3 [file mmc1.pdf]

iScience, Volume *26*

Supplemental information

Mutational burden of XPNPEP3 leads to defects in mitochondrial complex I and cilia in NPHPL1

Lingxiao Tong, Jia Rao, Chenxi Yang, Jie Xu, Yijun Lu, Yuchen Zhang, Xiaohui Cang, Shanshan Xie, Jianhua Mao, and Pingping Jiang

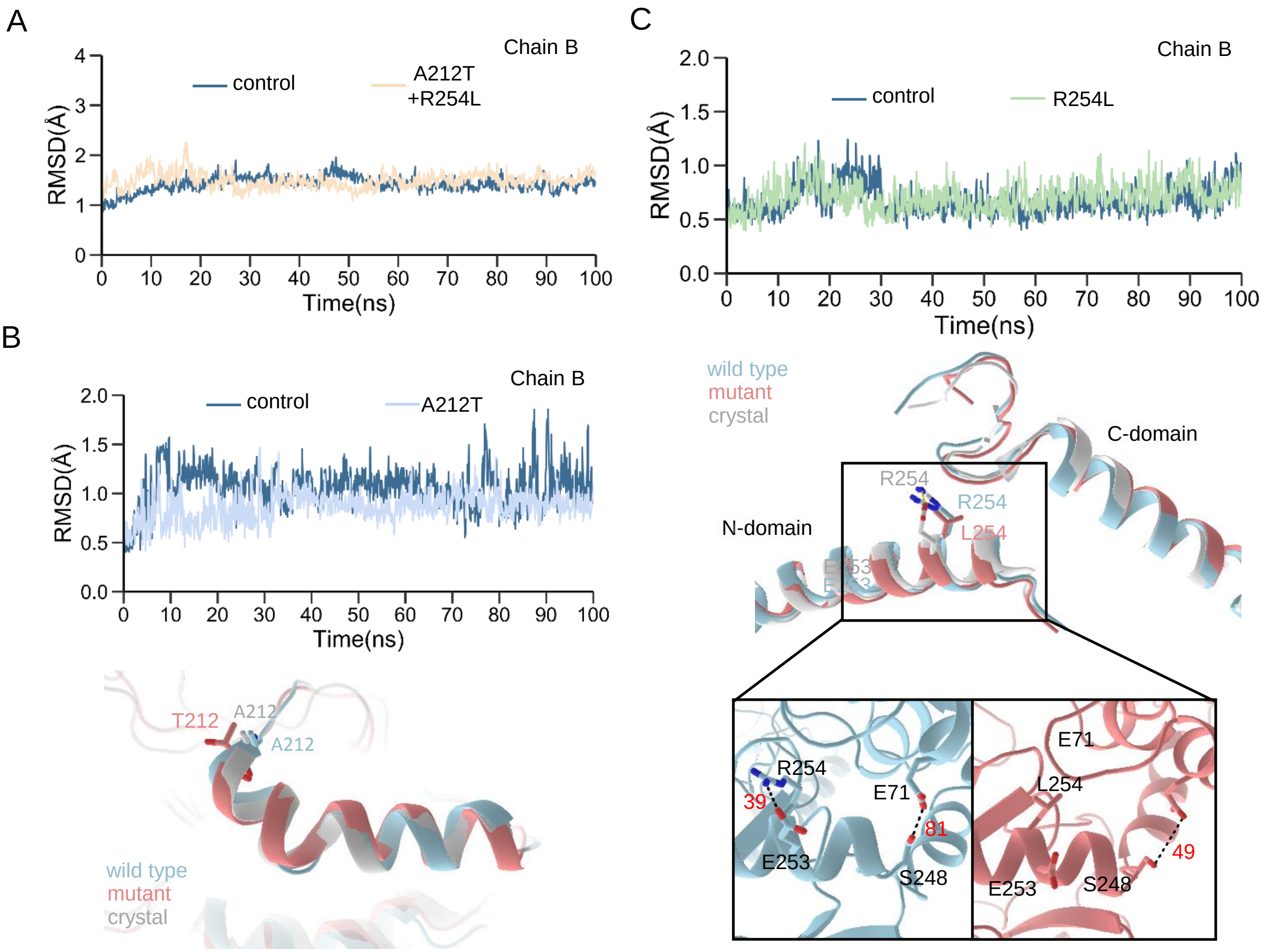

Fig.S1 Molecular dynamics simulations for chain B of XPNPEP3 (PDB:5X49), related to Figure 1.

(A) Time evolutions of the RMSD (root mean square deviation) between the wild-type and variant XPNPEP3m chain B with p.A212T and p.R254L of full-length XPNPEP3 chain B. (B) Time evolutions of the RMSD and structure diagrams of XPNPEP3 chain B residues 211-231. (C) Time evolutions of the RMSD and structure diagrams of XPNPEPm chain B residues 62-86 and 249-272. Dashed lines represent inter-domain interaction. No significant difference was detected in stability alteration between mutant and control.

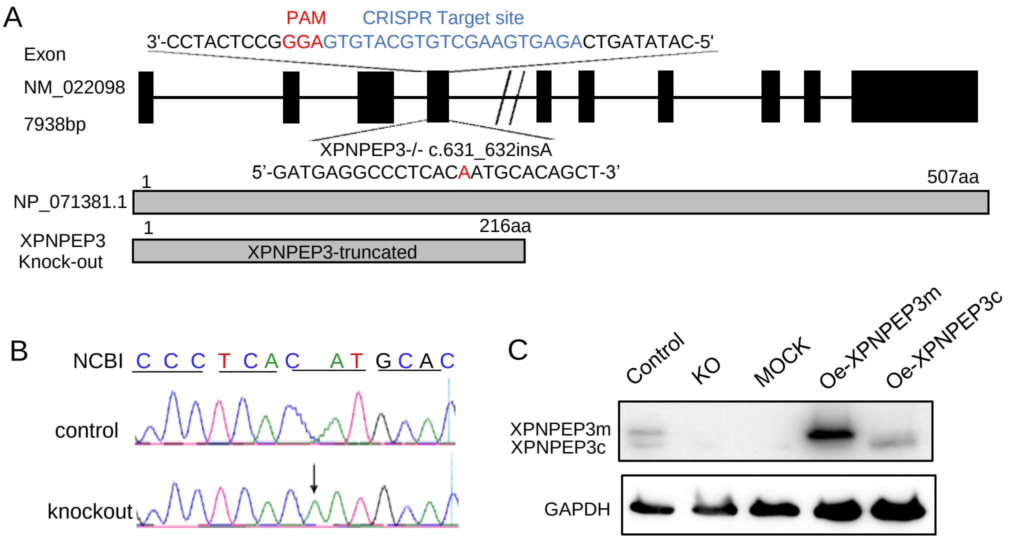

Fig S2. **Generation of XPNPEP3 knockout HK-2 cells using CRISPR/Cas9, related to Figure 2**. (A) schematic diagram for knock out XPNPEP3 in HK-2. A clone with a base A insertion between c.631 and c.632 (c.631_632 insA) in exon 4 was selected, which resulted in a truncated XPNPEP3 protein (p.His211Prof*5). (B) Identification with Sanger sequencing. (C) Western blotting assay for different HK-2 cell lines. KO, cells without XPNPEP3; MOCK, , exogenous empty vector in KO cells; Oe-XPNPEP3m, exogenous XPNPEP3m was overexpressed in KO cells. Oe-XPNPEP3c, exogenous XPNPEP3c was overexpressed in KO cells.

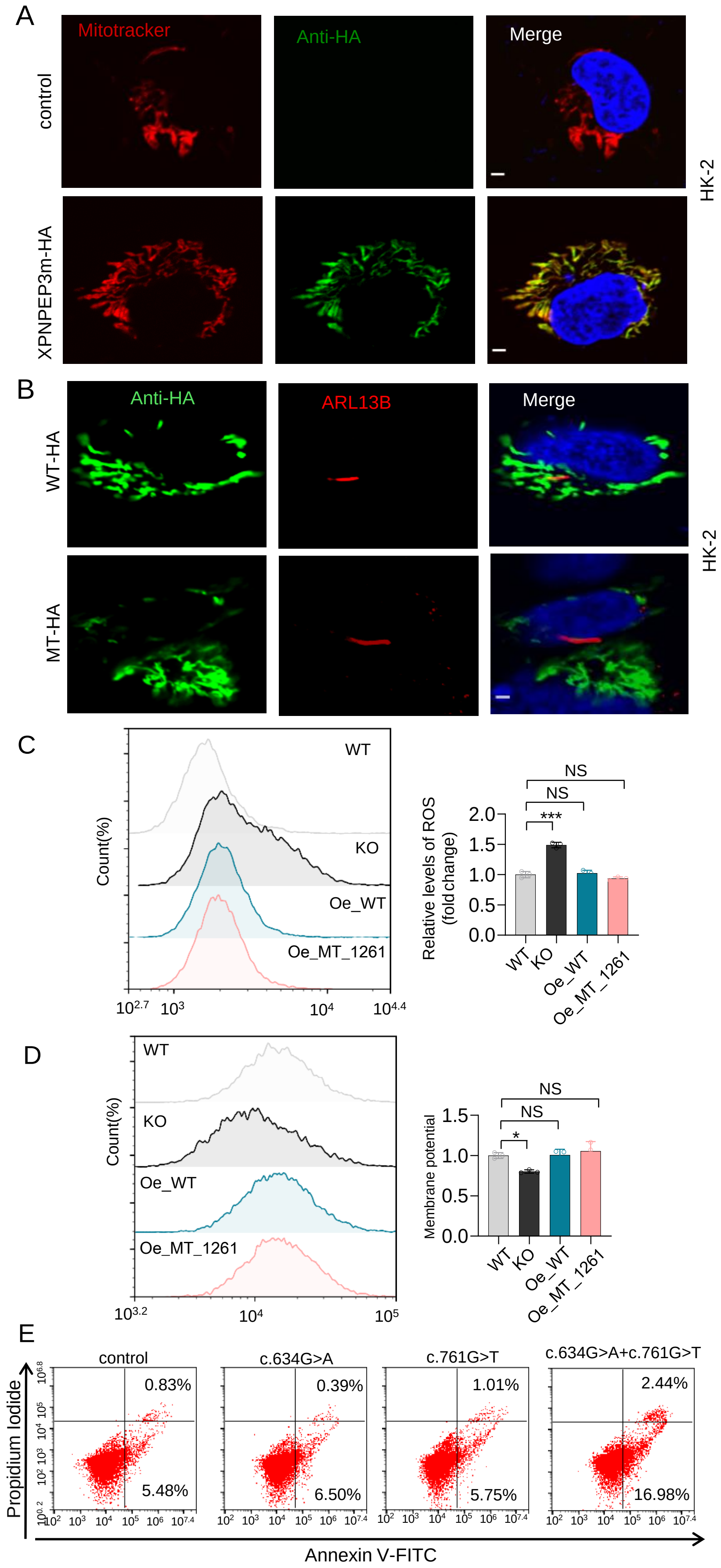

Fig S3 Localization of XPNPEP3 with mitochondria or with cilium, and its function on cell, related to Figure 3.

(A) Co-localization of exogenous XPNPEP3m and mitochondria in HK-2 cells . bar=2 μm. (B)Localization pattern of exogenous XPNPEP3m and primary cilium in HK-2 cells. WT, wild type XPNPEP3; MT, the mutant XPNPEP3. bar=2 μm. (C) Mitochondrial ROS in HK-2, measured by flow cytometry with MitoSOX (n=3). (D) The measurement of mitochondrial membrane potential (MMP) by flow cytometry with TMRM (n=3). (E) Apoptosis levels in lymphoblasts using annexinV and propidium iodide. Data is shown as the mean ± SD of triplicates at least.. One-way ANOVA was performed among four groups.*, $P < 0.05$, ***, $P < 0.001$, NS, not significant.

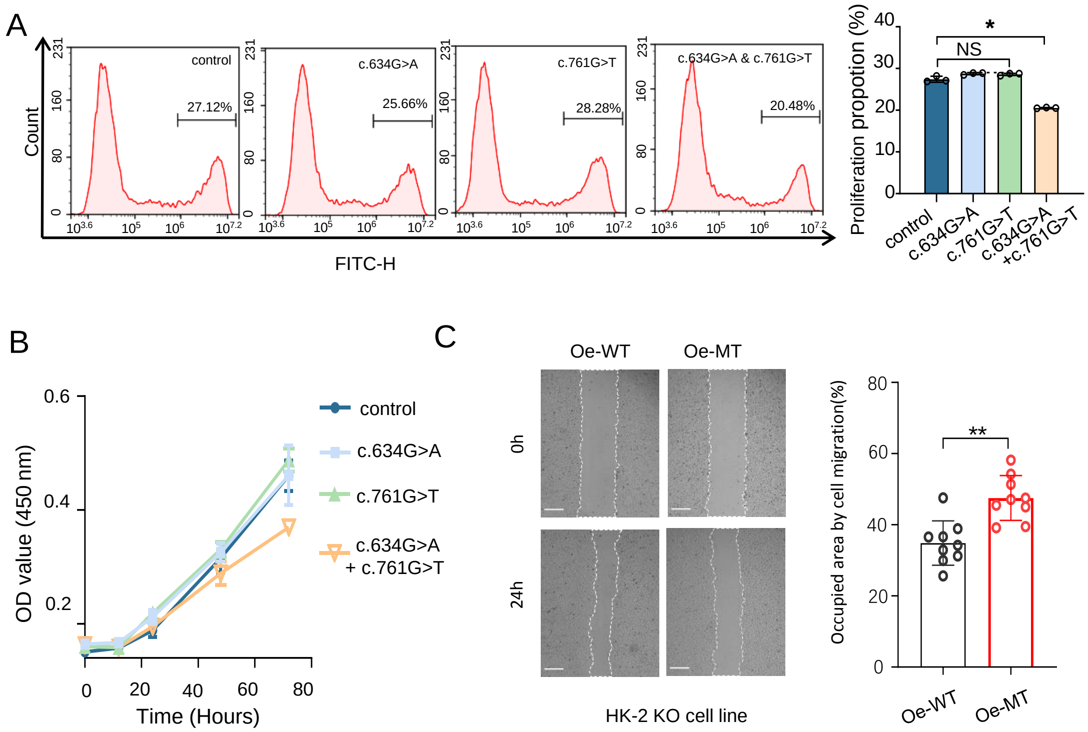

Fig S4. Impacts of XPNPEP3 on cell proliferation and migration, related to Figure 3.

(A) Cell proliferation assessed by EdU Flow Cytometry Assay kit in lymphoblasts. The proportion of EdU labeled cells was shown as histogram at left panel. (B) Cell proliferation analysis by CCK8 assay in lymphoblasts. (C) Wound closure analyzed at 24h after scratching.HK-2 cells preincubated with Mitomycin C (2μg/ml) for two hours. Quantitative analysis shown at left panel. Oe-WT, transfected wild type of XPNPEP3 back into KO cells; Oe-MT, transfected mutant XPNPEP3 (carrying c.634G>A and c.761G>T back into KO cells). Data was expressed as the means±SD of triplicates at least. Student's t-test was performed between two groups and one-way ANOVA was performed among four groups *, $P < 0.05$. **, $P < 0.01$. ***, $P < 0.001$. NS, not significant

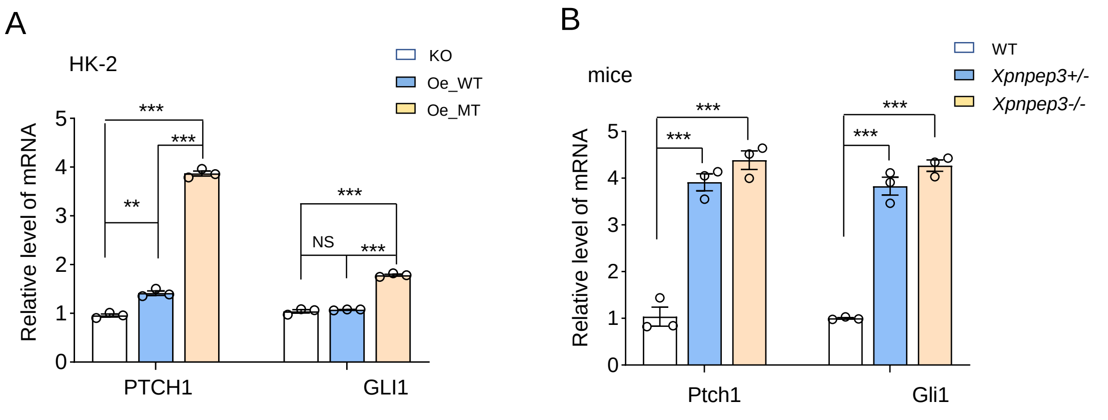

Fig S5 PTCH1 and GLI1 mRNA expression in cells and mice, related to Figure 5 & 6.

Reverse transcriptase quantitative PCR (RT–qPCR) analysis of PTCH1 and GLI1 mRNA in HK-2 cells. (A) and kidney tissues in mice(B). Data is shown as the mean ± SD of triplicates at least. One-way ANOVA was performed among three groups. *, $P < 0.05$; **, $P < 0.01$; ***, $P < 0.001$. NS, not significant.

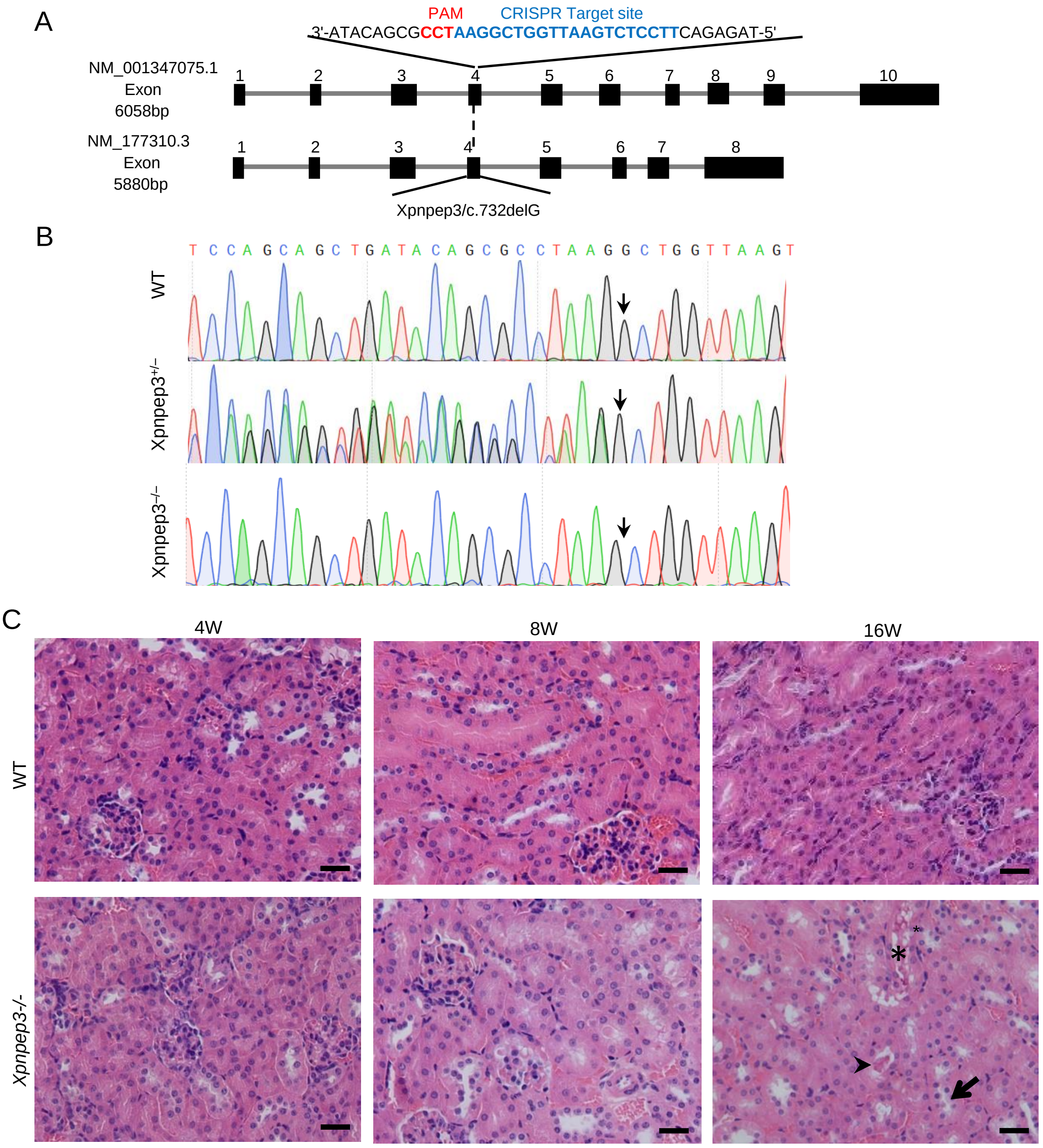

Fig S6. Generation of Xpnpep3-KO mice using CRISPR/Cas9, related to Figure 6.

(A) schematic diagram for knock out Xpnpep3 in mice, with a base deletion (c.732delG) in exon 4. (B) Identification with Sanger sequencing. (C) HE staining of kidney sections of mice at 4 weeks, 8weeks and 16 weeks mice. Arrow, tubular epithelial cell , Arrowhead, cast formation, Asterisk, vacuolar degeneration. Bar,50μm.

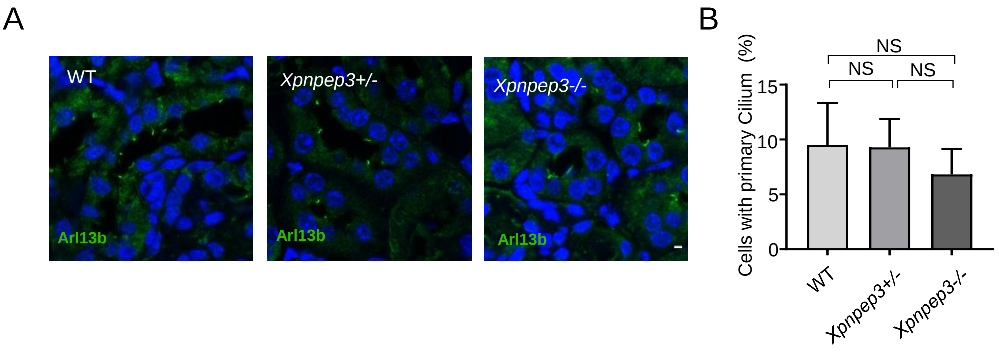

Figure S7 Amount of ciliary cells in kidney tubular epithelial cells in mice using antibodies against ciliary protein Arl13b. related to Figure 6.

(A) Immunofluorescence images, Bar, 1 µm. (B) Ratio of cells with primary cilium. Data is shown as the mean ± SD of triplicates at least. One-way ANOVA was performed among four groups. NS, not significant.

Table S1 The prediction of the variants on protein function of XPNPEP3, related to Figure 1

**Mutant**	**AA_change**	**Effect_refGene**	**Damaging_score**	**SIFT**	**Polyphen2_HDIV**	**PROVEAN**	**CADD_pred**	**MutationTaster**	**gnomAD**	**dbSNP**
c.-87C>T	-	-	-	-	-	-	-	-	0.00006	rs886057506
exon4:c.634G>A	p.A212T	nonsynonymous	0.04	Tolerable	Benign	Tolerable	Tolerable	Polymorphism	-	-
exon4:c.761G>T	p.R254L	nonsynonymous	0.13	Tolerable	Benign	Tolerable	Tolerable	Disease-causing	0.0001	rs373246644
exon9:c.1261C>G	p.H421D	nonsynonymous	0.91	Damaging	Probably_damaging	Damaging	Damaging	Disease-causing	4.061e-05	rs186524609

Ref sequence, XPNPEP3:NM_022098; -, absent.

Tabel S2. Primers used in this study, related to STAR Methods.

Primers		
XPNPEP3-634/761-F	AAAGAACACTGAAGTTTAGGGGC	
XPNPEP3-634/761-R	AAGCTAGATGCCATTCTGCGG	
XPNPEP3-(-87)-F	CAGCTCGGGGCATGACG	identification
XPNPEP3-(-87)-R	TTGCTACAGCGGGAACCAG	of variants
XPNPEP3-1261-F	GAGGTCGCAGTTTTTCAGGTG	
XPNPEP3-1261-R	GTAACACCTCTCCTTACCGGG	
18S-F	TAGAGGGACAAGTGGCGTTC	
18S-R	CGCTGAGCCAGTCAGTGT	
XPNPEP3-Total-F	CAAACCGATACTTAGGCCAGC	
XPNPEP3-Total-R	CCCTTGAGCTTCCTTCTGGAT	
XPNPEP3-Mito-F	TGTTCACAGCGAAGGTACTCC	
XPNPEP3-Mito-R	AGTTACCTCCCCTGGTCTGAG	
XPNPEP3-Pre-F	AATTGAACCTAACGAGGTGACAC	
XPNPEP3-Pre-R	AGAGCCATCGAATCCAGAGAC	
GAPDH-H-F	CAAGGTCATCCATGACAACTTTG	
GAPDH-H-R	GTCCACCACCCTGTTGCTGTAG	
GLI1-H-F	CCCGGAGTGCAGTCAAGTT	qRT-PCR
GLI1-H-R	CCAGAGATGGGCTCATGGTG	
PTCH1-H-F	GCCGCGTTAATCCCAATTCC	
PTCH1-H-R	GCAGGGGCTTGTAAAACAGC	
Gapdh-M-F	GCAATGCATCCTGCACCACCA	
Gapdh-M-R	TTCCAGAGGGGCCATCCACA	
Gli1-M-F	CTCGACCTGCAAACCGTAATC	
Gli1-M-R	TCCTAAAGAAGGGCTCATGGTA	
Ptch1-M-F	TCACAACCACGACCCTGGACGA	
Ptch1-M-R	CAGTCCCAGCGCAGCATGGTTA	
XPNPEP3-Mus-F	GCTGAGACGAACATGGTTTGG	mice
XPNPEP3-Mus-R	AATTGAACCTAACGAGGTGACAC	identification
XPNPEP3m- probe-F	taatacgactcactataggg<br>AGTTACCTCCCCTGGTCTGAG	
XPNPEP3m-probe-R	CTCAGACCAGGGGAGGTAACT<br>ccctatagtgagtcgtatta	RNA Fish Probe
XPNPEP3-c.634G>A-F	AGGCCCTCACATACACAGCTTCACTC<br>TGACTATATGC	
XPNPEP3-c.634G>A-R	GTGAAGCTGTGTATGTGAGGGCCTC<br>ATCCAGTCATAC	
XPNPEP3-c.761G>T-F	AGAAATTGAACTAATGCAGATTGCT<br>GGGAAGCTGA	site-directed<br>mutagenesis
XPNPEP3-c.761G>T-R	CAGCAATCTGCATTAGTTCAATTTCT<br>GCAGGAGACT	

XPNPEP3-c.1261C>G-F	TCCTCATGATGTTGGCCACTACCTC GGGATGG
XPNPEP3-c.1261C>G-R	AGTGGCCAACATCATGAGGACAGTA TTTTCGAGCAGC

Tabel S3 Key reagent and resources in experiments, related to STAR Methods.

REAGENT or RESOURCE	SOURCE	IDENTIFIER	Dilution Ratio
Antibodies			
XPNPEP1	Abcam	Cat#ab235324	1：1000 (WB)
XPNPEP2	Proteintech	Cat#25945-1-AP	1：1000 (WB)
AQP1	Proteintech	Cat#66805-1-Ig	1：500 (IF)
KIM1	Abcam	Cat#ab228973	1：500 (IF)
NGAL	Proteintech	Cat#26991-1-AP	1：500 (IF)
XPNPEP3	Genetex	Cat#GTX105541	1：1000 (WB)
GAPDH	Abcam	Cat# ab8245	1：20000 (WB)
TOM20	Abclonal	Cat#A19403	1：1000 (WB)
UPF1	Proteintech	Cat#23379-1-AP	1：1000 (WB)
DCP2	Abclonal	Cat#A8282	1：1000 (WB)
G3BP1	Proteintech	Cat#13057-2-AP	1：1000 (WB)
ELAVL1	Proteintech	Cat#11910-1-AP	1：1000 (WB)<br>1：200 (IF)
KSRP	Proteintech	Cat#55409-1-AP	1：1000 (WB)
β-actin	Proteintech	Cat#66009-1-Ig	1：2000 (WB)
Total OXPHOS Rodent WB Antibody Cocktail	Abcam	Cat#ab110413	1：500 (WB)
COX-IV	Abcam	Cat#ab16056	1：1000 (WB)
NDUFB8	Proteintech	Cat#14794-1-AP	1：1000 (WB)
NDUFB4	Abclonal	Cat#A13820	1：1000 (WB)
NDUFB7	Proteintech	Cat#14912-1-AP	1：1000 (WB)
NDUFA13	Abclonal	Cat#17257-1-AP	1：1000 (WB)
NDUFA8	Abclonal	Cat#A12118	1：1000 (WB)
NDUFB11	Proteintech	Cat#16720-1-AP	1：1000 (WB)
NDUFA3	Proteintech	Cat#17257-1-AP	1：1000 (WB)
NDUFS1	Proteintech	Cat#12444-1-AP	1：1000 (WB)
ATP5a	Abcam	Cat#ab176569	1：1000 (WB)
UQCRC2	Proteintech	Cat#14742-1-AP	1：2000 (WB)
COX5a	Abcam	Cat#ab181226	1：1000 (WB)
SDHB	Abcam	Cat#ab178423	1：1000 (WB)
Cytochrosome c	Abclonal	Cat#A4912	1：1000 (WB)
Caspase3	CST	Cat#9662	1：1000 (WB)
PARP	CST	Cat#9532	1：1000 (WB)
PTCH1	Abclonal	Cat#A0826	1：1000 (WB)
SMO	Proteintech	Cat#66851-1-Ig	1：1000 (WB)
GLI1	Proteintech	Cat#66905-1-Ig	1：5000 (WB)
Anti-HA	Abclonal	Cat#AE008	1：2000 (WB)
Calbindin	Proteintech	Cat#66394-1-Ig	1：500(IF)
ARL13B	Proteintech	Cat#17711-1-AP	1：500 (IF)

Complex I Immunocapture	abcam	Cat#ab109798	1：10 (IP)
Goat anti mouse IgG(H+L) (HRP)	Beyotime	Cat# A0216	1：5000 (WB)
Goat anti rabbit IgG(H+L) (HRP)	Beyotime	Cat# A0208	1：5000 (WB)
Alexa Fluor 488 goat anti-mouse IgG	Abcam	Cat#ab150113	1：500 (IF)
Alexa Fluor 594 goat anti-rabbit IgG	Abcam	Cat#ab150080	1：500 (IF)
Anti-IgG	Proteintech	Cat#B900620	1：200(IP)
Critical Commercial Assays			
MitoSOX™ Red Mitochondrial Superoxide Indicator, for live-cell imaging	Invitrogen	Cat# M36008	
JC-10 Mitochondrial Membrane Potential Assay Kit (Flow Cytometry)	Abcam	Cat#ab112133	
CellTiter-Glo Luminescent Cell Viability Assay	Promega	Cat#G7571	
RiboTM RNAmax-T7 Transcription Kit	RiboBio	Cat#C11080-1	
Reverse transcription kit	Takara	Cat#6110A	
Cell apoptosis assay kit	Beyotime	Cat#C1062L	
EDU flow-cytometry kit	Sangon Biotech	Cat#E607204	
Chemicals			
DAPI	Sigma	Cat#D9542	
Mitotracker	CST	Cat#9082	
Mitomycin C	Selleck	Cat#BIO-000001	
CCK8	Yeasen	Cat#40203ES60	
Trizol reagent	Sangon	Cat#B511311	
Hieff Trans transfection reagent	Yeasen	Cat#40802ES03	
Puromycin	Gibco	Cat#A1113802	
DDM	MedChemExpress	Cat#HY-128974	
Actinomycin D	Sigma	Cat#A9415	
H-Lys(Abz)-Pro-Pro-pNA	Bachem	Cat#4027668	
FCCP	Sigma	Cat# C2920-10MG	
NBT	Merck	Cat#298-83-9	
NADH	Merck	Cat#606-68-8	
DAB	Merck	Cat#91-95-2	
cytochrome c	Merck	Cat#C3483	
