# Supplementary material for: Mutational burden of XPNPEP3 leads to defects in mitochondrial complex I and cilia in NPHPL1
Source: iScience. 2023 Jul 23;26(8):107446. doi: 10.1016/j.isci.2023.107446 (PMC10432713; doi:10.1016/j.isci.2023.107446)
Supplement: Datas S1–S19 [file mmc2.pdf]

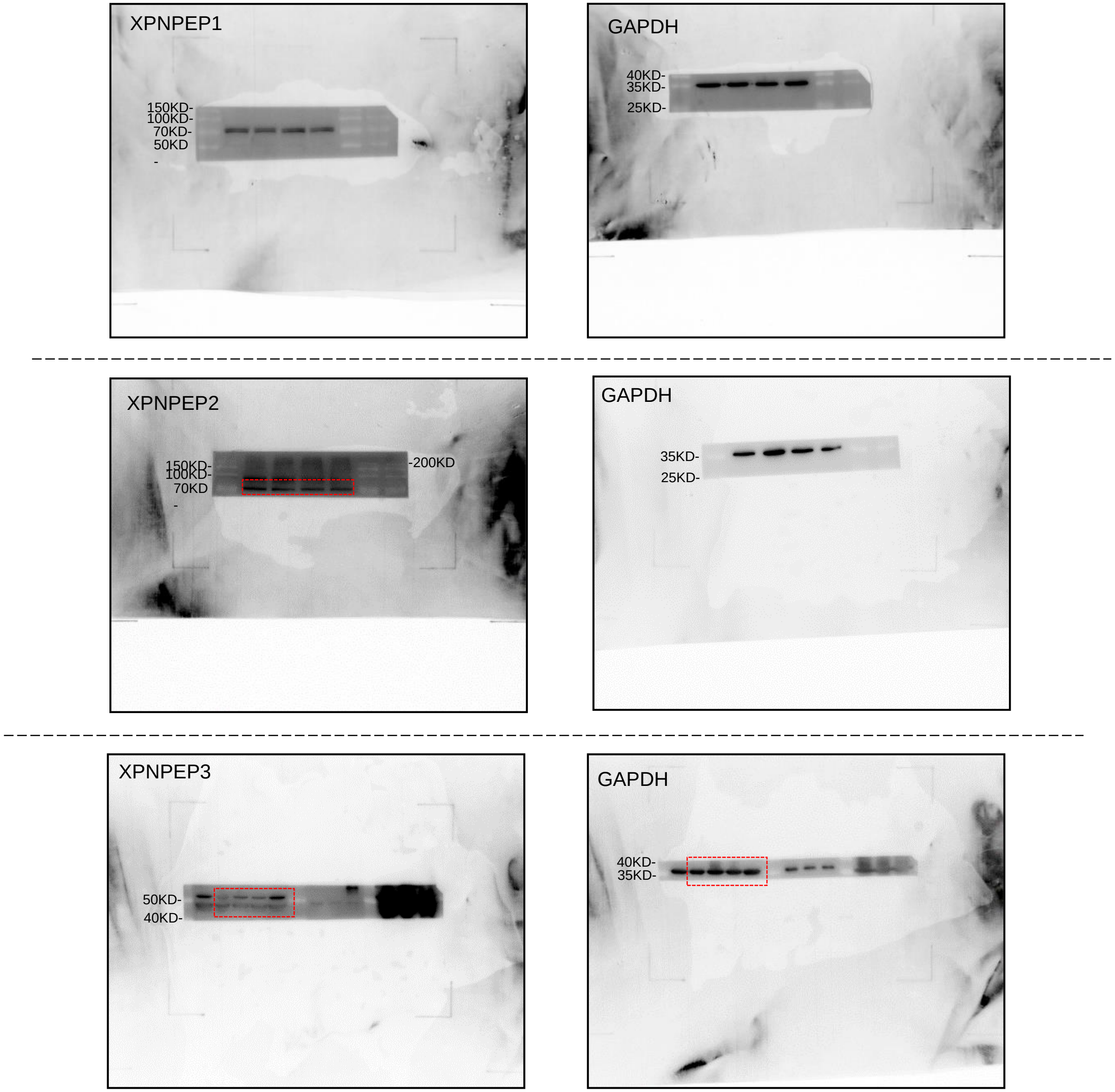

Data S1.Western blot analysis of XPNPEP1, XPNPEP2, and XPNPEP3 in lymphoblast cell lines in family A and an age-matched control to patient A, related to Figure 2.

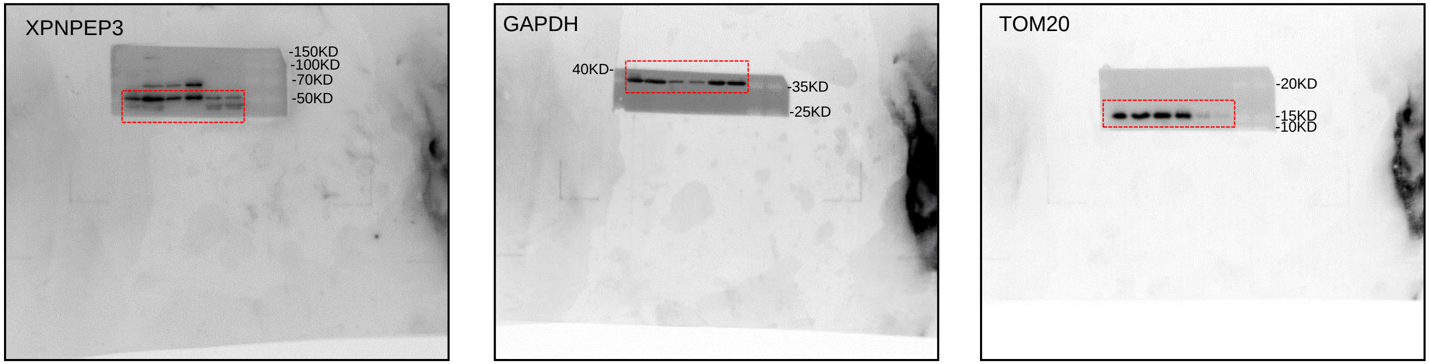

Data S2. Subcellular expression of XPNPEP3 by WB with anti-XPNPEP3, related to Figure 2.

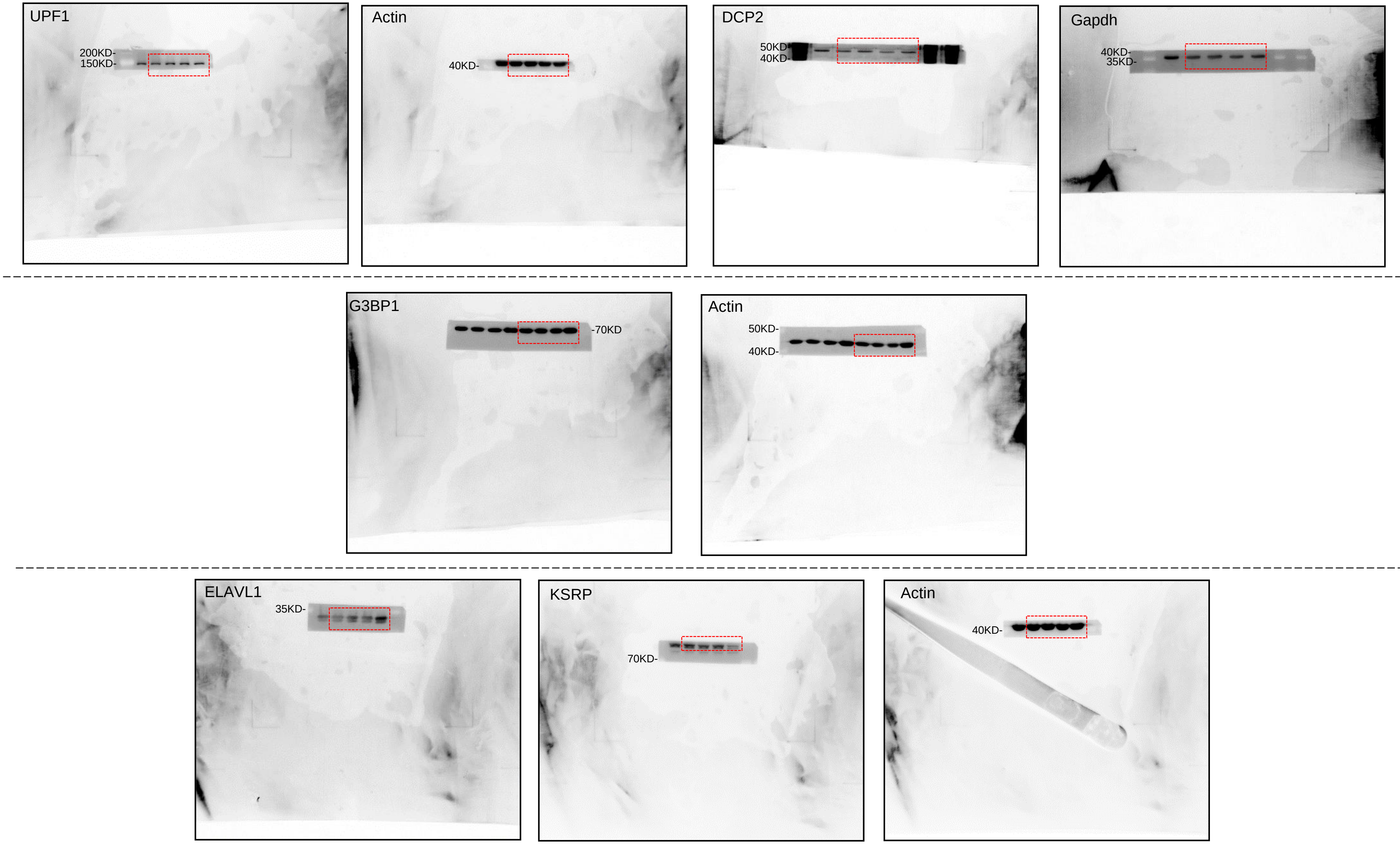

Data S3. Protein levels of UPF1, DCP2, G3BP1 ,ELAVL1 and KSRP detected by Western Blotting, related to Figure 2.

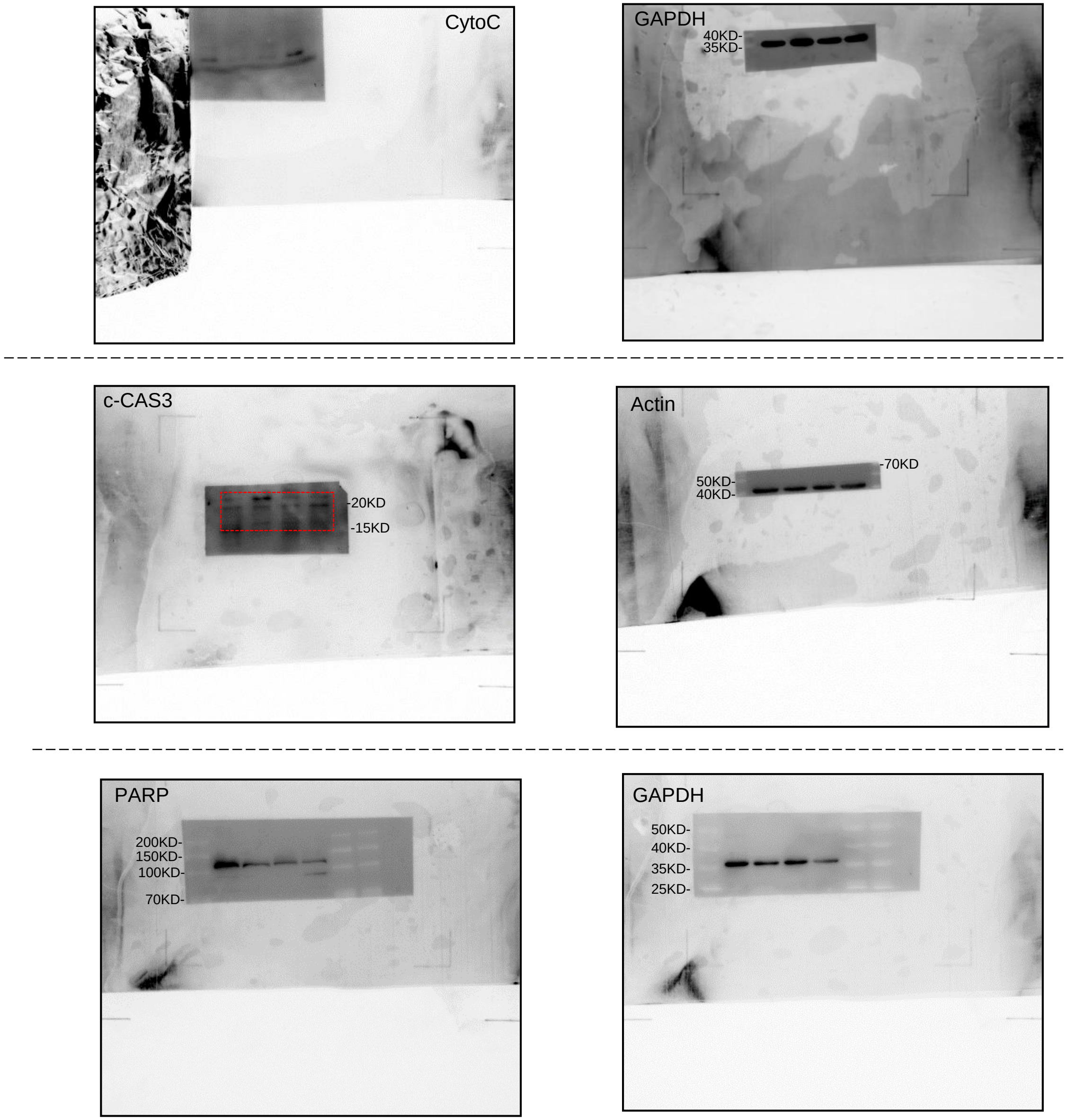

Data S4. Western blot analysis of apoptosis-associated proteins with antibodies against Cytc , c-CAS3 , PARP, c-PARP , related to Figure 3.

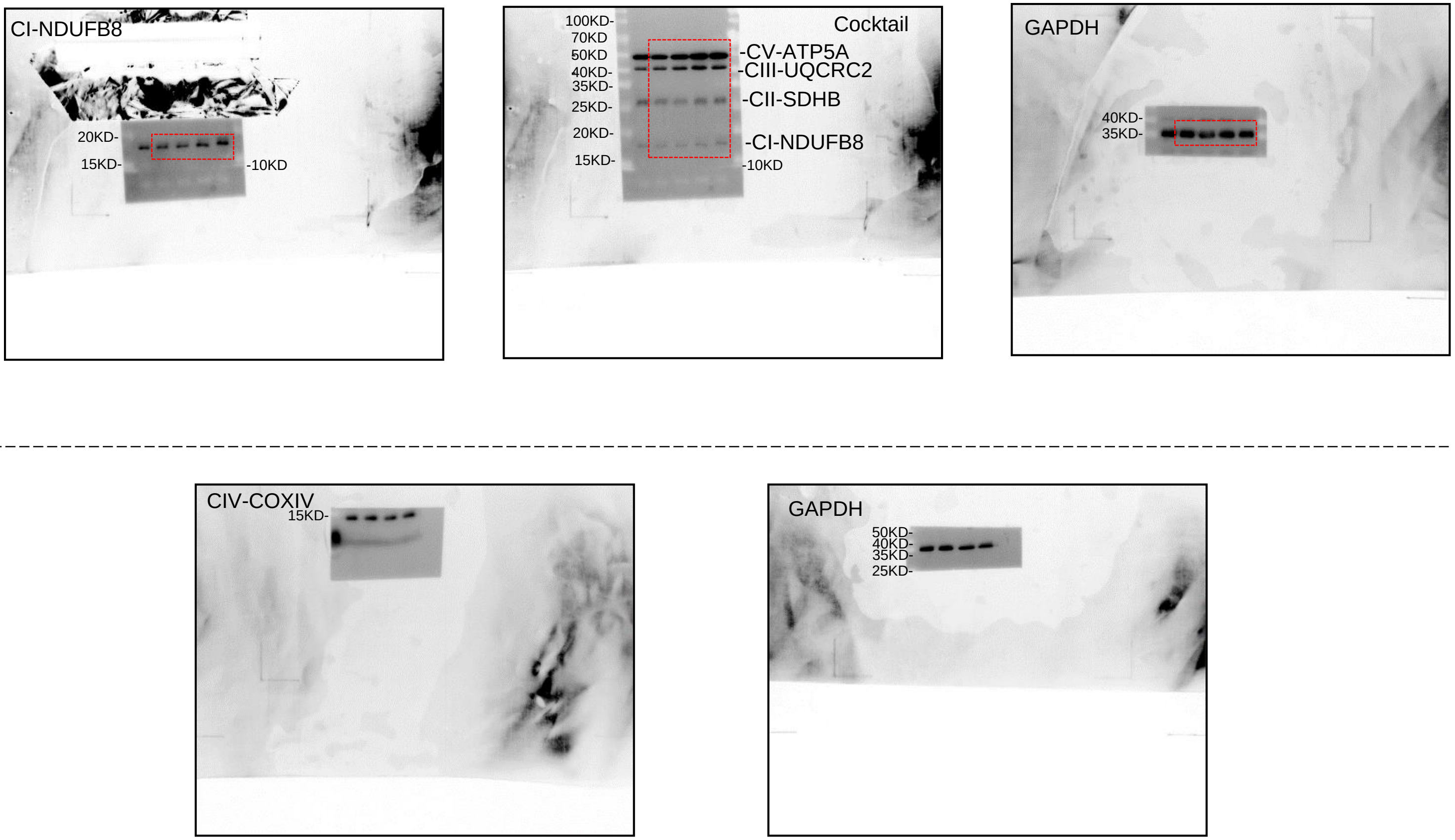

Data S5. Western blot analysis of subunits of respiratory complexes I-V in lymphoblasts, related to Figure 4.

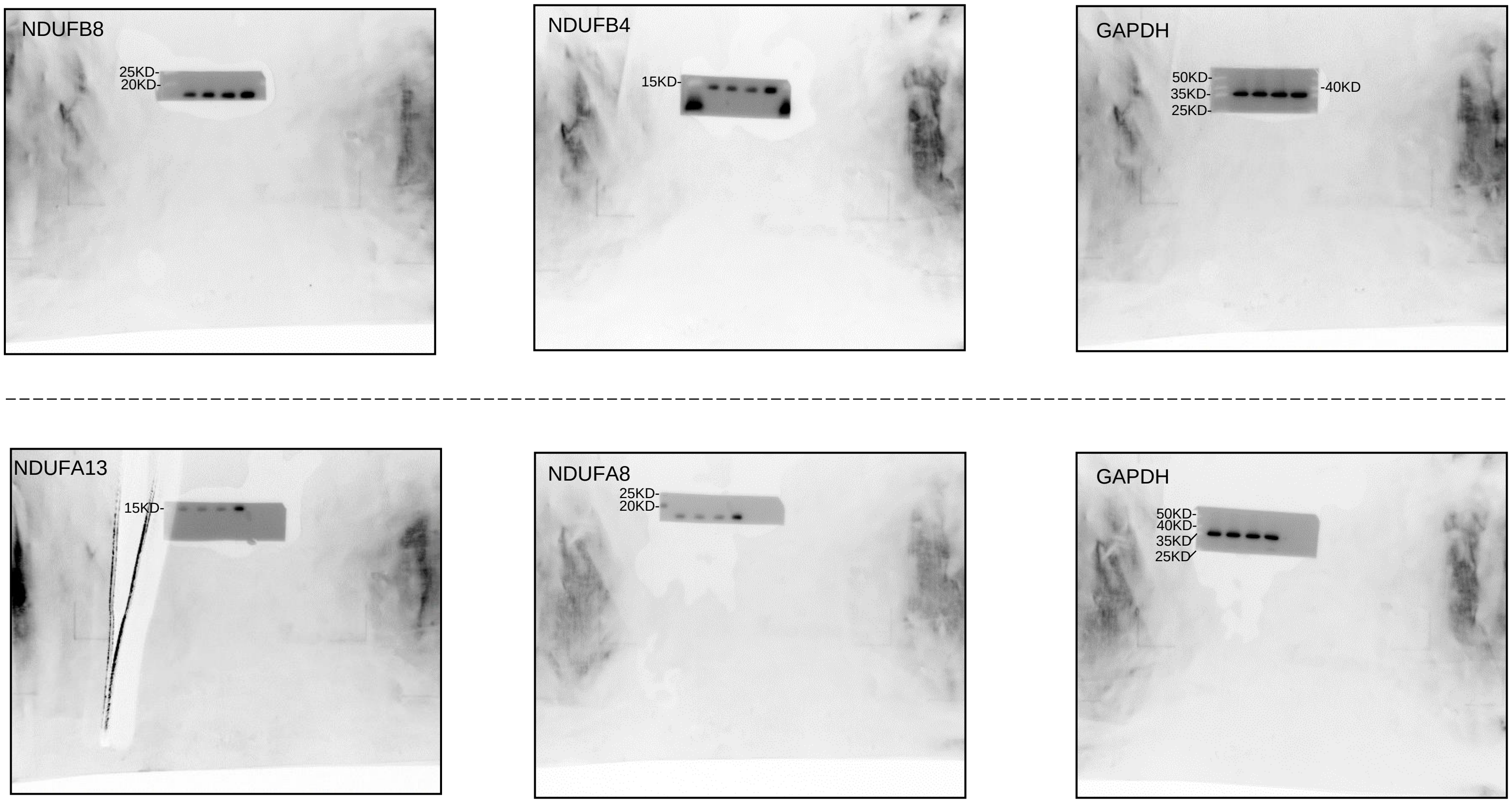

Data S6. Western blot analyses of subunits of complex I in lymphoblasts, related to Figure 4.

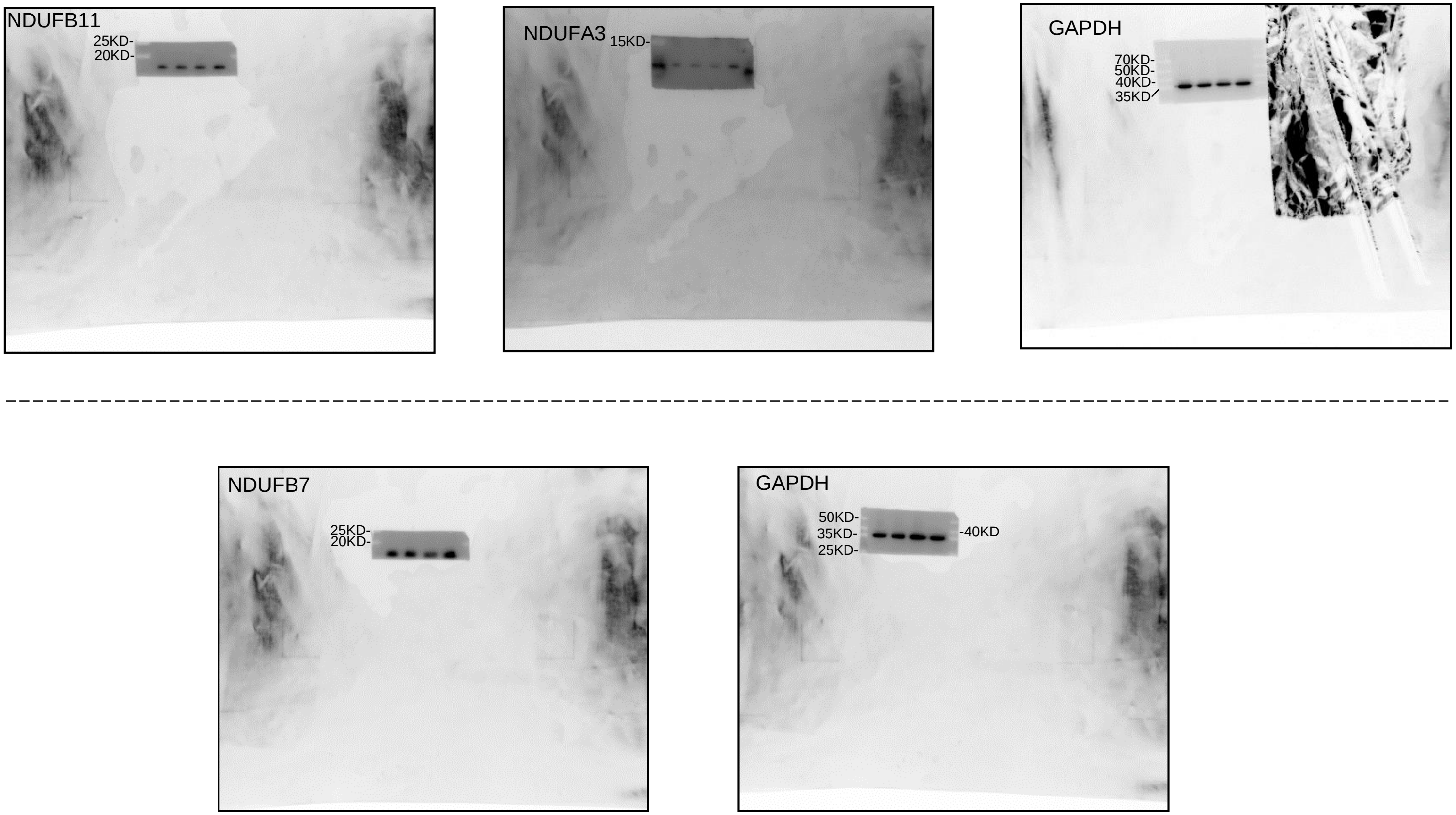

Data S7. Western blot analyses of subunits of complex I in lymphoblasts, related to Figure 4.

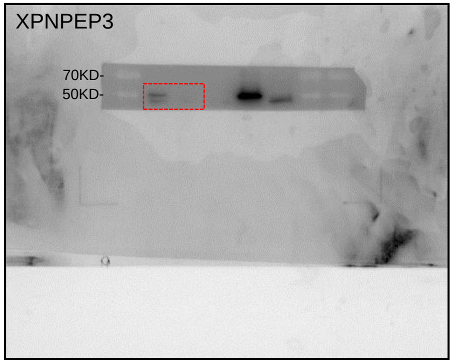

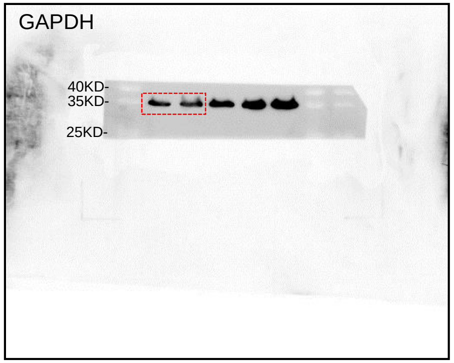

Data S8. Western blot analyses of XPNPEP3 in HK-2 cell lines, related to Figure 4.

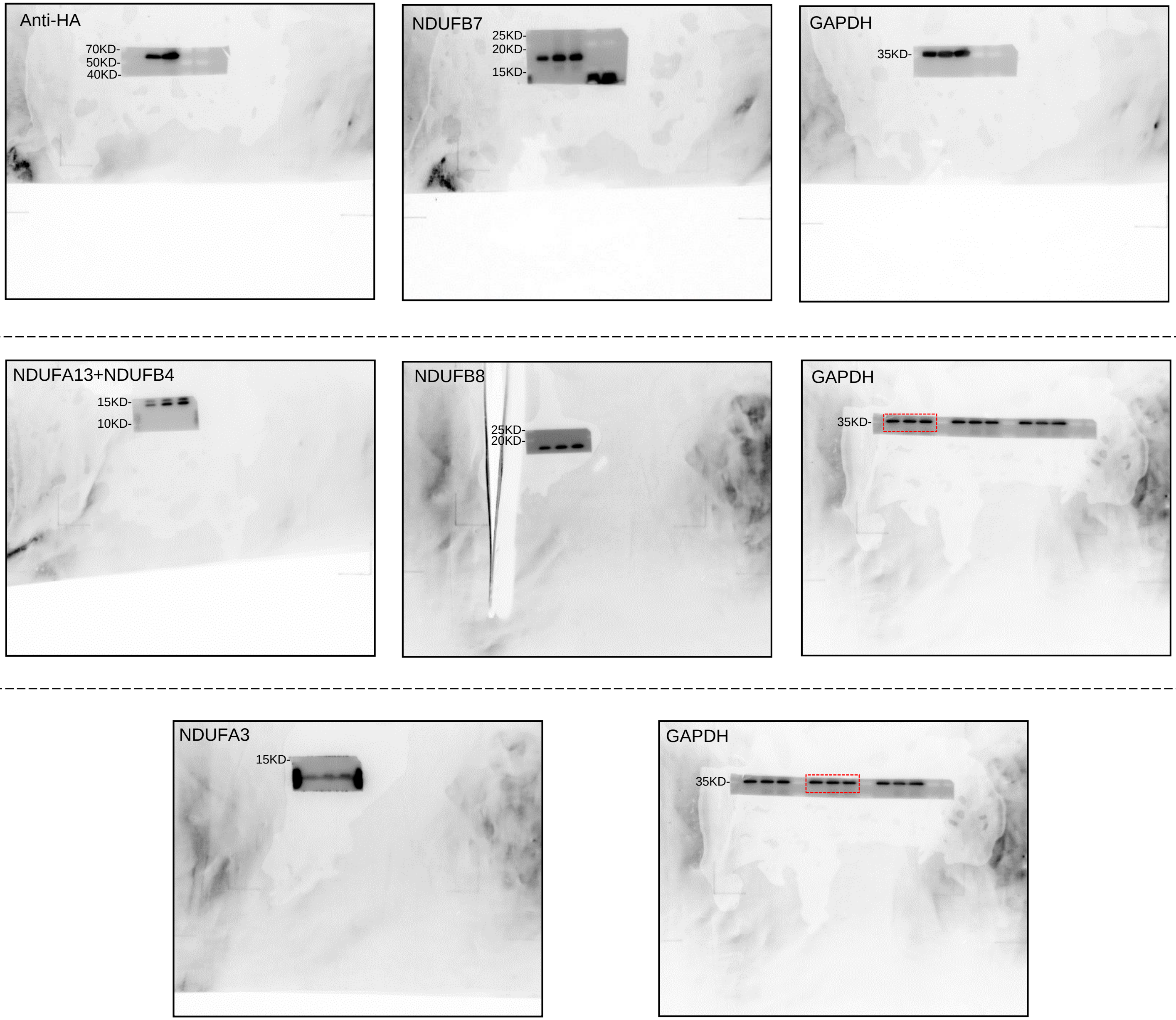

Data S9. Western blot analyses of subunits of complex I in HK-2 cell lines, related to Figure 4.

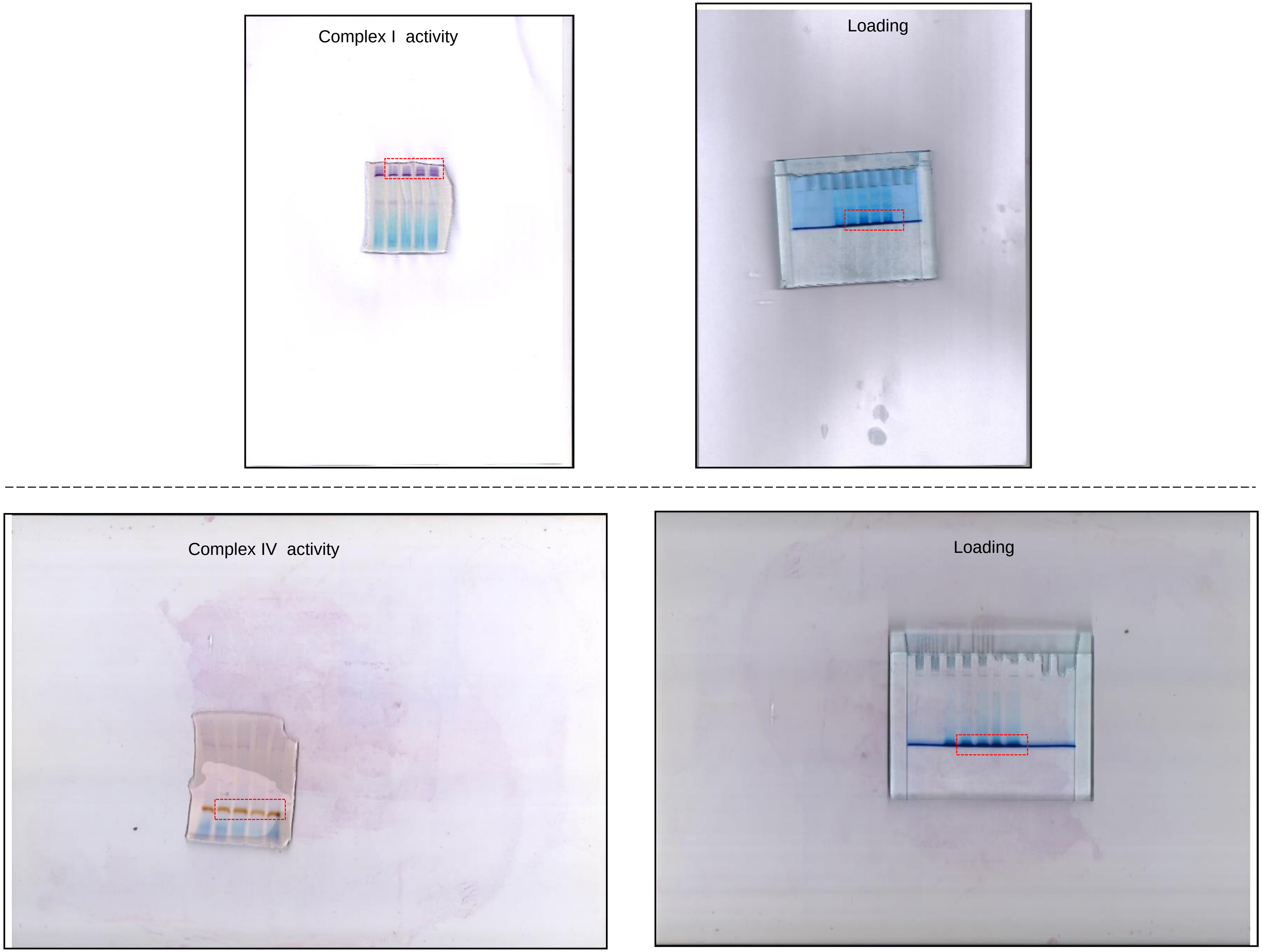

Data S10. In-gel activity of complexes I and IV in lymphoblasts by native PAGE, related to Figure 4.

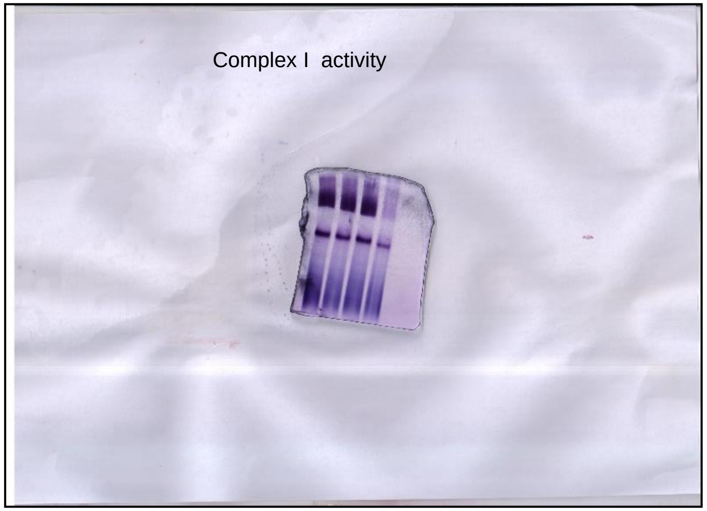

Loading

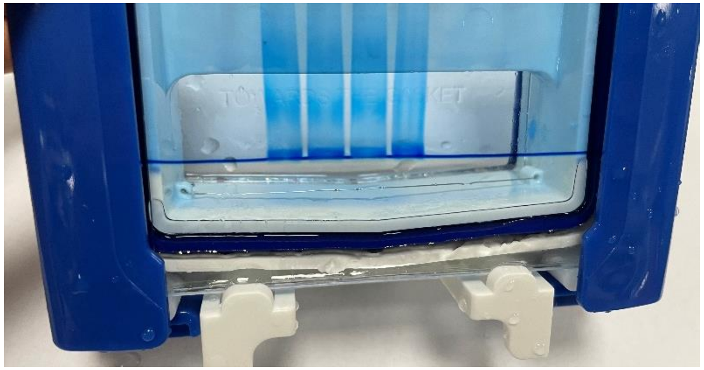

Data S11. In-gel activity of complexes I in HK-2 cell lines, related to Figure 4.

BN-PAGE

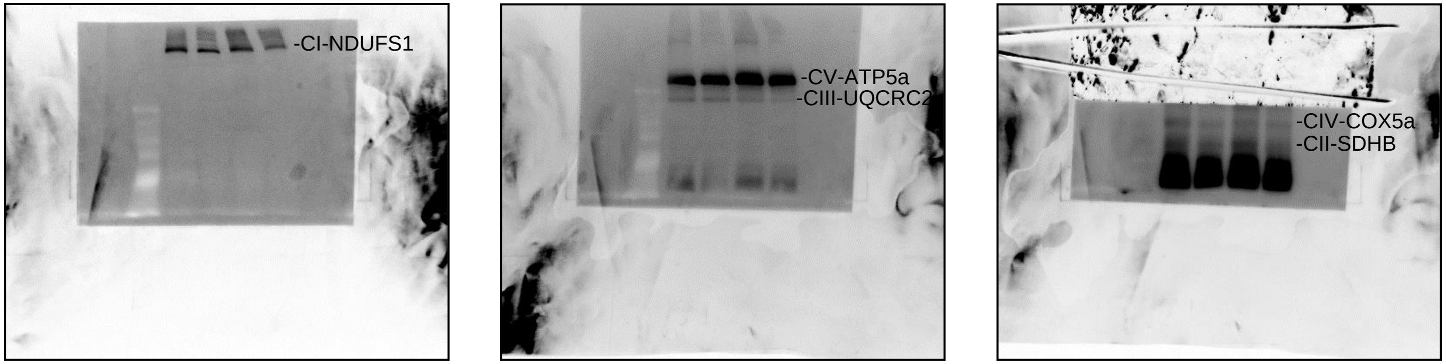

WB

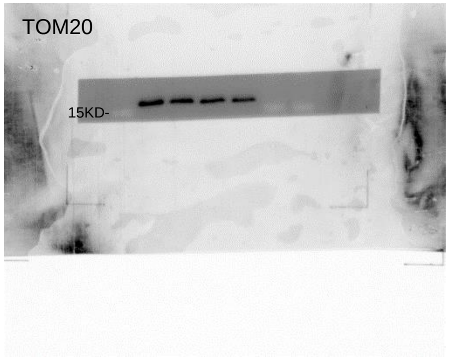

Data S12. Stability of fully assembled complex I in lymphoblasts by BN-PAGE analysis, hybridized with antibody cocktail specific for subunits of OXPHOS complex (up) and with TOM20 as a loading control (down), related to Figure 4.

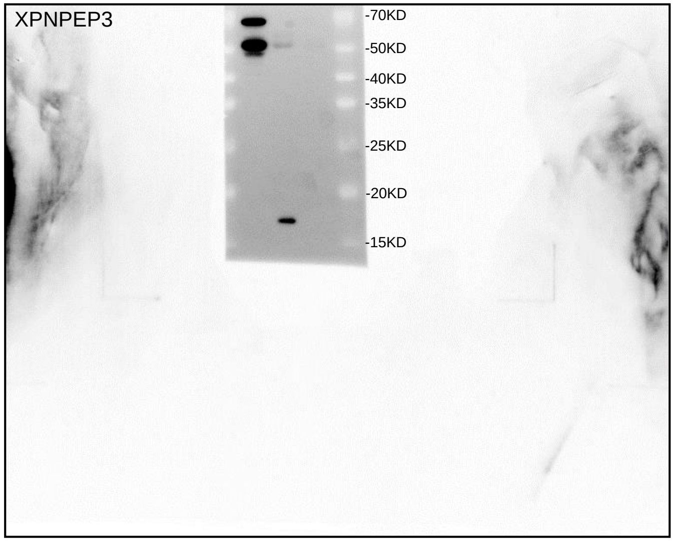

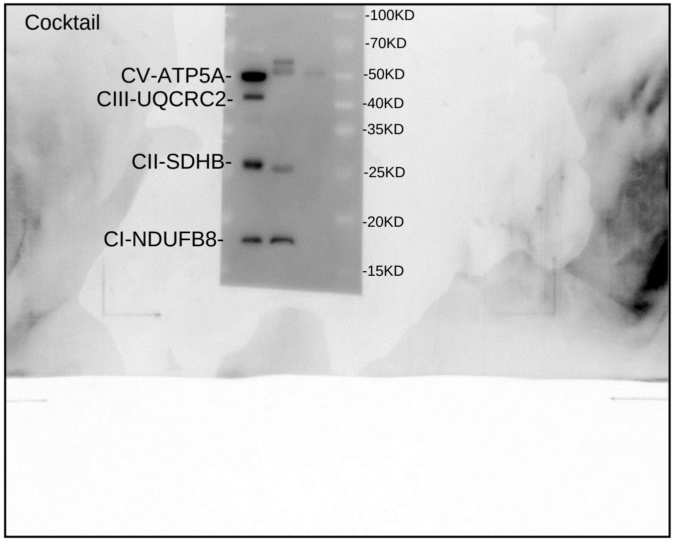

Data S13. Immunoprecipitation by complex I capture antibody and immunoblotted with anti-XPNPEP3, UQCRC2 for complex III, and NDUFB8 for complex I, related to Figure 4.

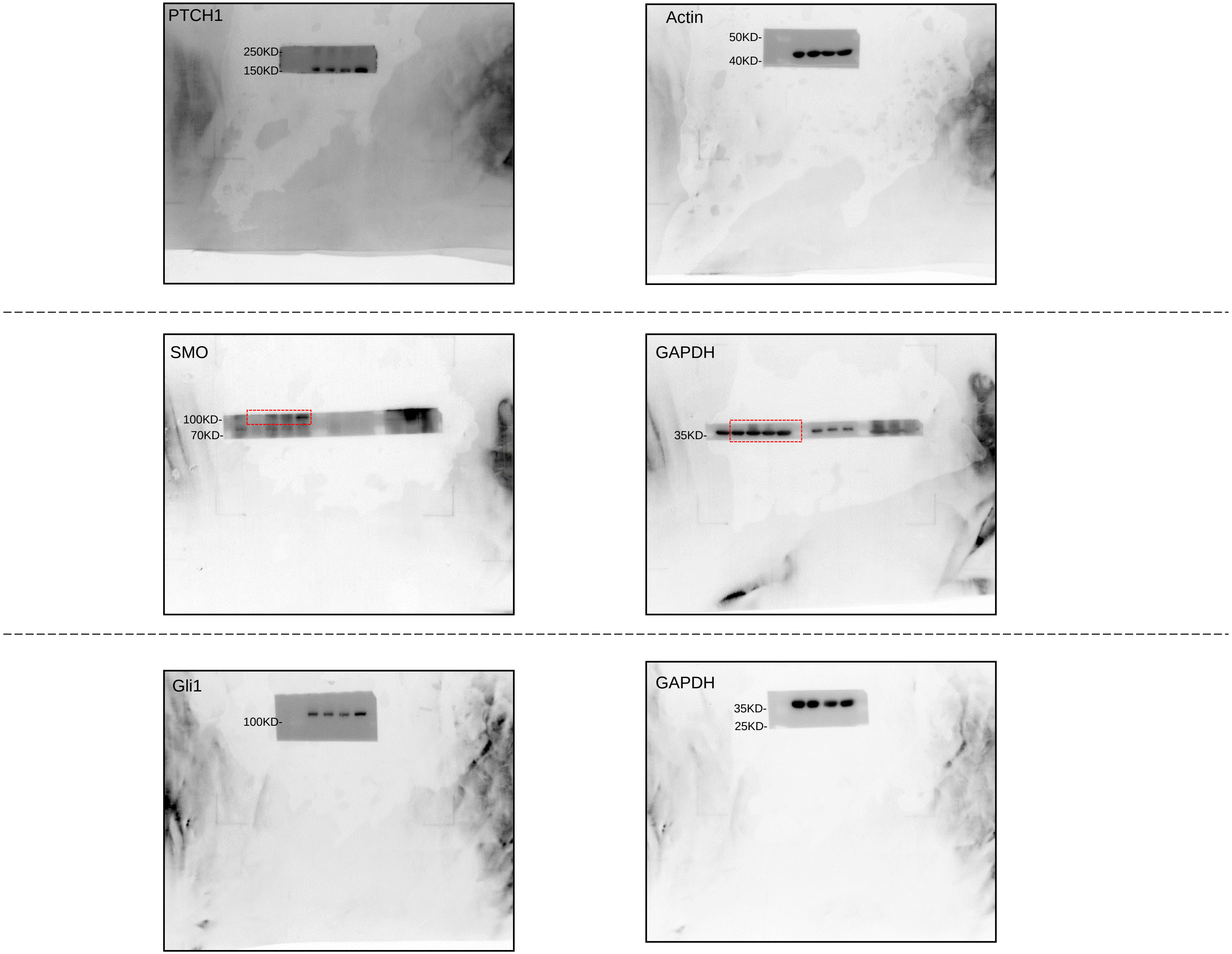

Data S14. Western blot analysis for ciliary function in lymphocytes with antibodies against proteins involved in Hh signaling pathway, related to Figure 5.

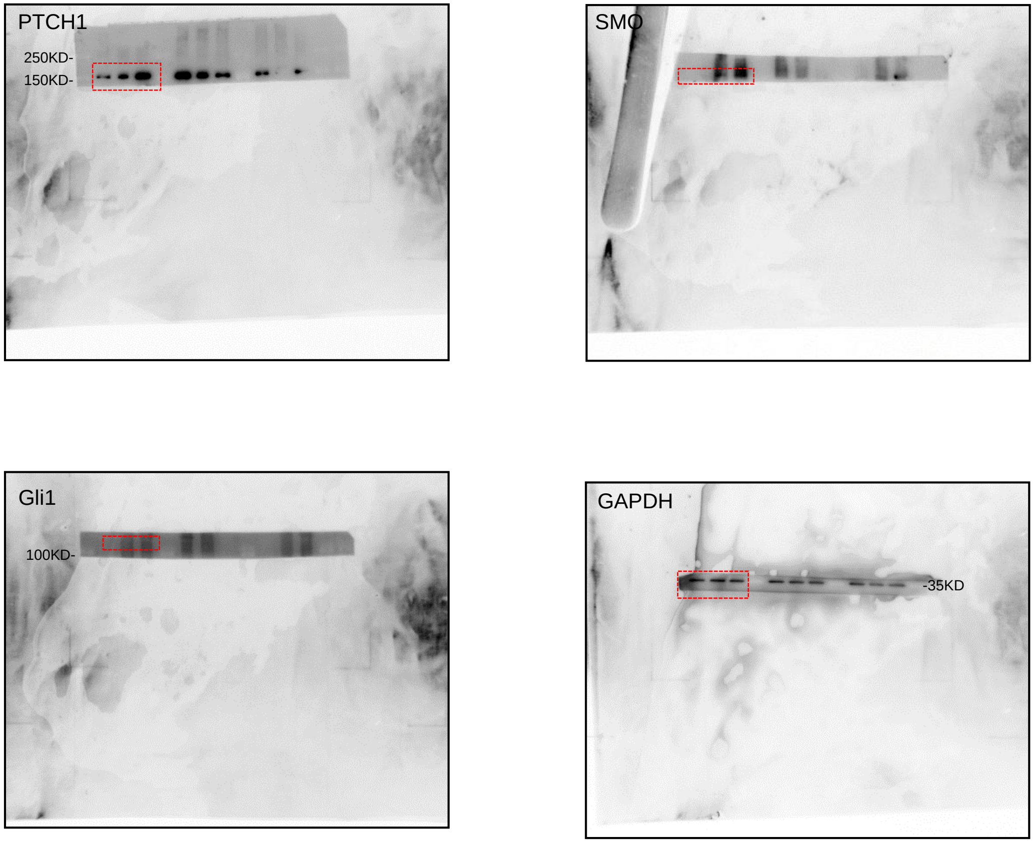

Data S15. Western blot analysis for ciliary function in HK-2 cell lines with antibodies against proteins involved in Hh signaling pathway , related to Figure 5.

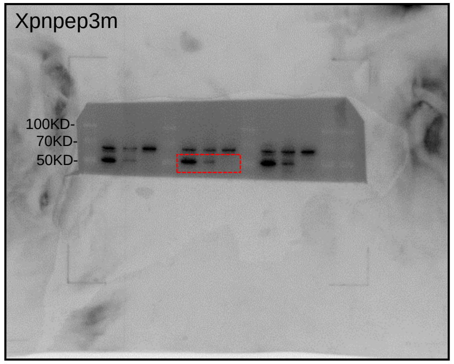

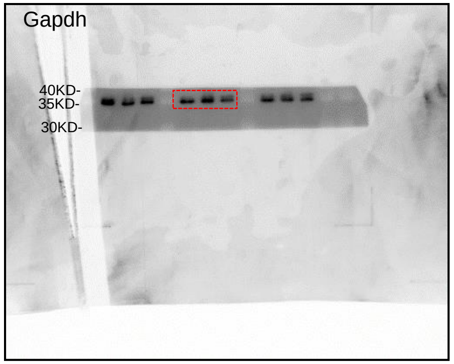

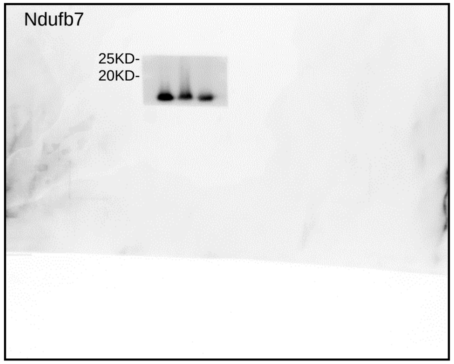

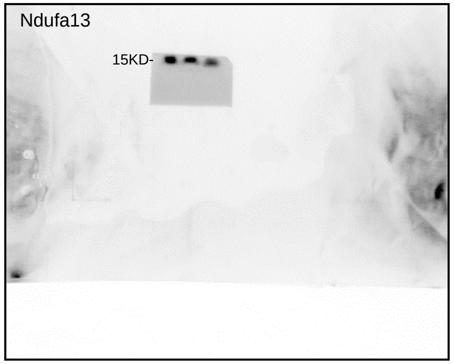

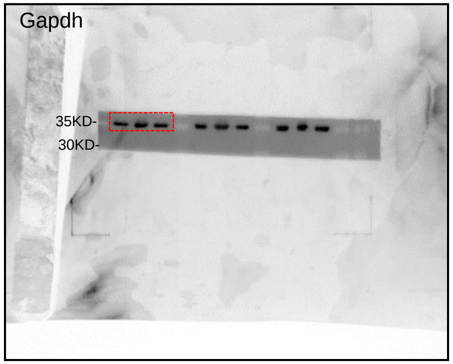

Data S16. Western blot analysis for subunits of mitochondria complex I from kidney tissues, related to Figure 6.

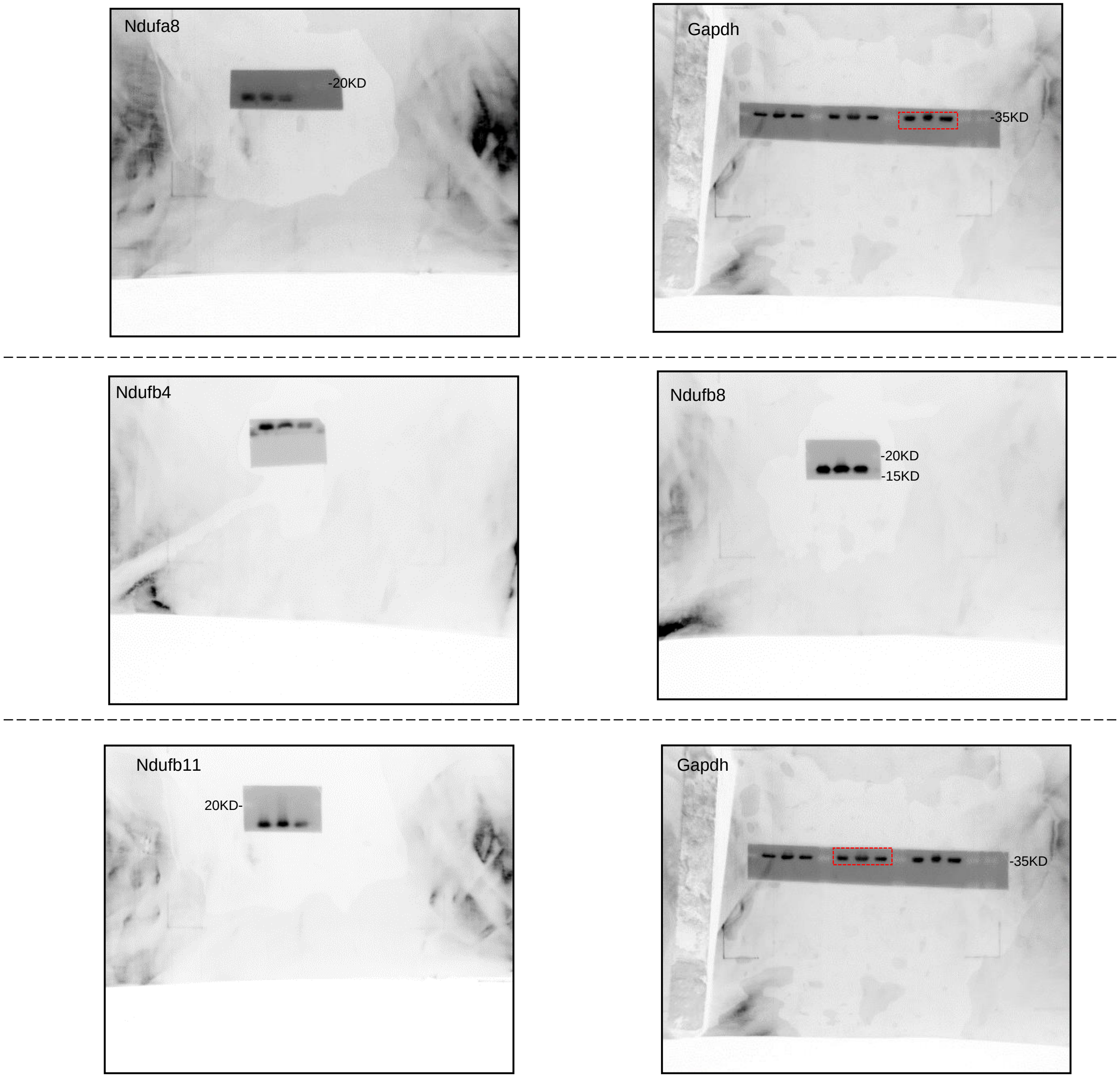

Data S17. Western blot analysis for subunits of mitochondria complex I from kidney tissues, related to Figure 6.

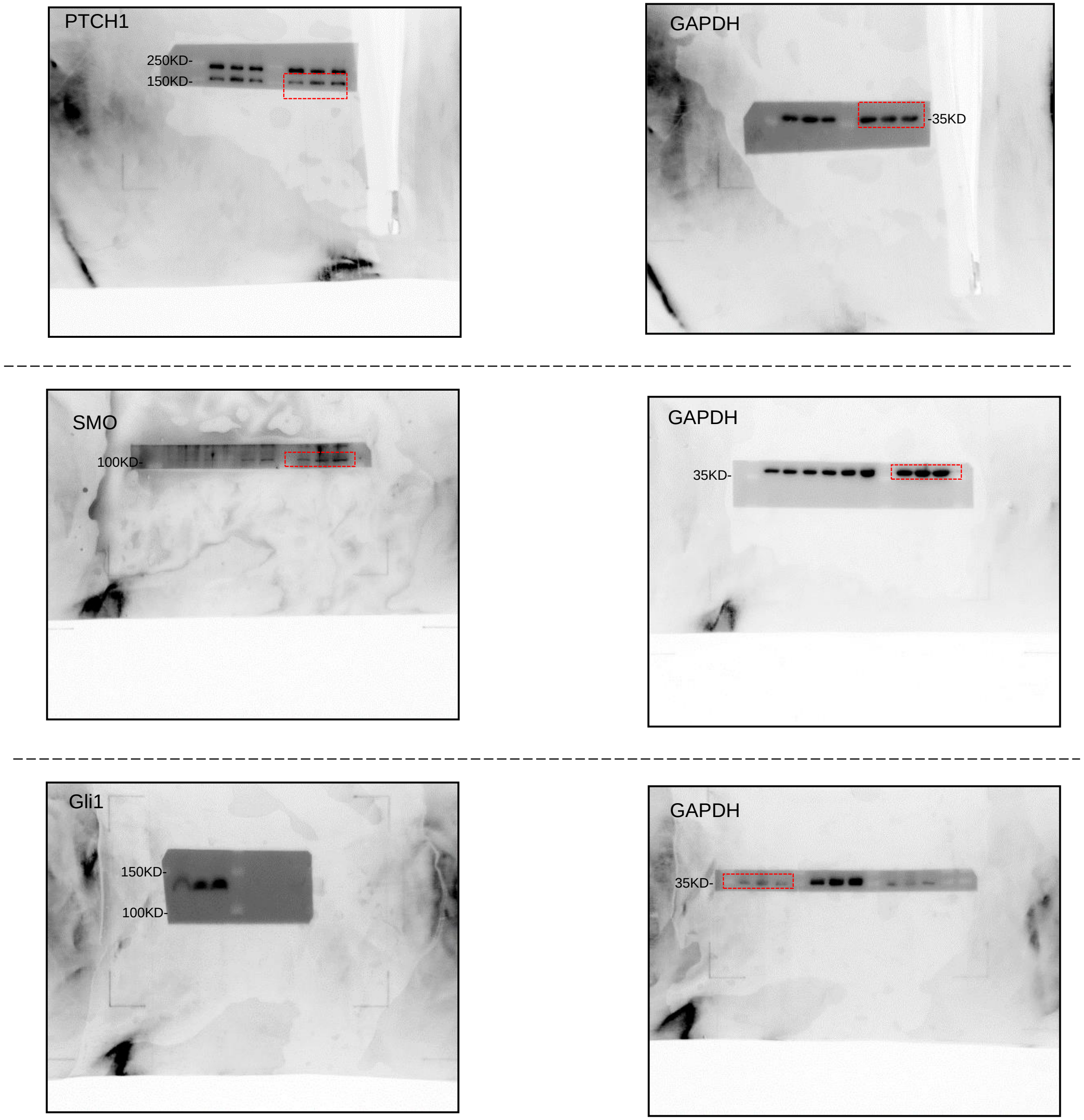

Data S18. Western blot analysis for Hh pathway protein Ptch1, Smo, and Gli1 in renal tissues, related to Figure 6.

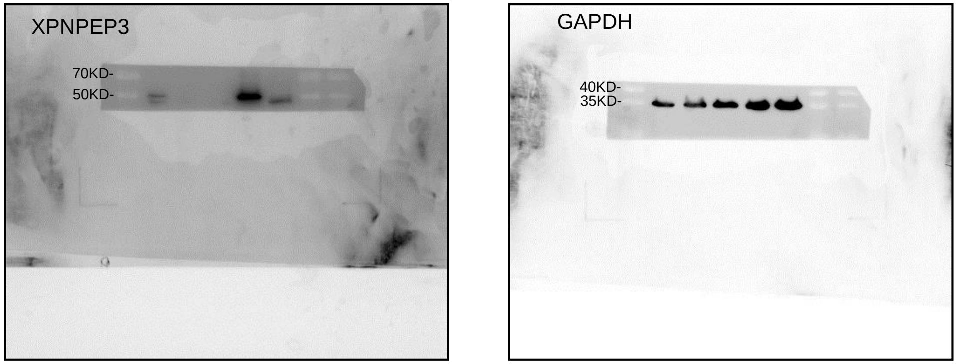

Data S19. Western blotting assay for different HK-2 cell lines, related to Figure S2.
